# The genetic landscape of primary malignant melanoma of the cervix using integrated bioinformatics analysis and whole-exome sequencing

**DOI:** 10.3389/fonc.2025.1597153

**Published:** 2025-12-17

**Authors:** Jialiu Zhou, Tian Xu, Chujun Chen, Yi Ping

**Affiliations:** Department of Obstetrics and Gynecology, Second Hospital of Shanxi Medical University, Taiyuan, China

**Keywords:** whole exome sequencing, bioinformatics analysis, microarray, primary malignant melanoma of the cervix, protein-protein interaction

## Abstract

**Background:**

Primary malignant melanoma in the cervix (PMMC) currently has no standardized therapy. Its pathogenesis remains unclear, and the prognosis is poor. Given the high mortality of PMMC, its causes and pathogenic mechanisms need to be unraveled, and novel biomarkers must be identified.

**Methods:**

To further understand the genomic characteristics of PMMC, whole-exome sequencing was performed on the cancerous and adjacent tissues from three patients with PMMC, and PMMC-related tumor-susceptibility genes, mutation spectrum, mutation characteristics, driver genes, and high-frequency tumor mutations were analyzed. Concurrently, SNP and copy number variation (CNV) data from melanoma patients in the COSMIC and TCGA datasets were analyzed.Key protein interactions were further validated in vitro using co-immunoprecipitation assays.

**Results:**

The results showed that C>T/G>A is predominant in PMMC, with mutation characteristics more closely resembling those of SBS5 and SBS40, though not completely matching those of melanoma. Comparison with the COSMIC and TCGA databases revealed overlapping genes between PMMC and melanoma, such as TP53, AHNAK2, and PMMC, as well as previously unreported mutated genes such as AKT3, SMYD4, ATAD3A, SIRPB1, XKR6, etc. In vitro experiments and co-immunoprecipitation validation revealed that P53 interacts with AKT3, SMYD4, and ATAD3A, forming a network potentially involved in the pathogenesis of PMMC. Finally, integration of significantly mutated genes and CNV analysis revealed that copy number gains may regulate the expression of SIRPB1, AHNAK2, and XKR6, thereby activating the nuclear factor kappa B (NFkB) and protein kinase (AKT) signaling pathways, which could further promote the occurrence and development of PMMC.

**Conclusion:**

This study shows that while PMMC has similarities with other melanomas, it also harbors unique mutant genes. These findings provide a basis for exploring PMMC-related molecular markers and developing personalized diagnosis and treatment plans for patients with PMMC.

## 1 Introduction

Mucosal melanoma is a rare malignant melanoma (MM), accounting for only 1.4% of all melanomas, although MM is commonly found in the skin and mucous membranes (1, 2). Melanoma arises from melanocytes, which are embryonic neural crest cells that migrate to the skin, eyes, mouth, and genital mucosa during embryonic development (3). Primary malignant melanoma in the cervix (PMMC) is a MM with aggressive activity, which is extremely rare. Less than 2% of all MM cases occur in the female genital tract (4–6).

Female reproductive MM mainly occurs in the vulva and vagina. Cervical MM has the same origin as the skin primary MM. Pusceddu et al. (7) reported that, from 1889 to 2009, only 78 cases of PMMC, all reported retrospectively, were published in the literature. Due to the low incidence of PMMC, there are few reports about clinical outcomes and clinicopathological findings. Clinical reports have indicated that a high degree of malignancy is observed in cervical and vaginal melanoma and that relapse is common (8). The curative effects of operation, radiotherapy, and chemotherapy are poor, with a very poor prognosis and a 5-year survival rate of <25% (9). Diagnosing melanoma may be difficult, particularly in cases lacking pigment, due to challenges in distinguishing it from adenocarcinoma, mixed Müllerian tumors, leiomyosarcoma, rhabdomyosarcoma, and poorly differentiated squamous cell carcinoma. Currently, clinical diagnosis is mainly based on clinical manifestations, physical examination, endoscopy, and imaging examination. Pathological examination is the gold standard for diagnosis and staging (10). Thus, potential markers for diagnosis and highly efficient treatments need to be urgently identified.

In recent years, an increasing number of studies using microarrays and high-throughput sequencing have been conducted to explore common cancer biomarkers and the direct drugs for the diagnosis, therapy, and prognosis of cancer (11–14). Whole-exome sequencing (WES), which has been proven to be a useful approach for identifying new biomarkers in other diseases, enables the exploration of genetic alterations in PMMC. Additionally, recent cancer research has employed integrated bioinformatics methods to overcome inconsistent or limited results arising from the use of different technological platforms or small sample sizes, thereby uncovering substantial biological information (15–17).

In this study, we collected data on three patients with PMMC, performed WES, and then examined single-nucleotide polymorphisms/insertions-deletions (SNP/InDel), somatic single-nucleotide variation (SNV)/InDel, and somatic copy number variation (CNV). We further used Cancer Gene Census (CGC), Bert Vogelstein, SMG127, and a comprehensive analysis of tumor susceptibility genes and known driver genes available in the database, and high-frequency gene pathway enrichment analysis. Comparison with melanoma data in the COSMIC and TCGA databases revealed pathogenic genes different from skin melanoma (SKCM). Furthermore, frequently mutated genes and CNV-affected gene signaling pathways were validated at the cellular level and in clinical samples, providing a foundation for subsequent diagnosis and treatment of MM.

## 2 Materials and methods

### 2.1 Patient cohort and sample processing

#### 2.1.1 Sample collection and ethical approval

This study was conducted in strict accordance with the ethical principles outlined in the Declaration of Helsinki. Formal approval for all procedures involving human subjects was granted by the Institutional Review Board (Ethics Committee) of the Second Hospital of Shanxi Medical University (No. 2019YX260). Written informed consent was obtained from all participating individuals before their inclusion in the study.

The study cohort comprised three patients diagnosed with PMMC. A pair of formalin-fixed paraffin-embedded (FFPE) pathological tissue blocks was procured from each patient at the Department of Pathology at the Second Hospital of Shanxi Medical University in Taiyuan, China. Each pair consisted of one sample of the primary cancerous tumor tissue and one sample of matched, histologically confirmed adjacent non-cancerous (para-cancerous) tissue, yielding a total of six samples for analysis. **Supplementary Table 1** shows detailed clinical characteristics for the three groups, including age, height, weight, BMI, HPV infection status, TNM stage, and FIGO stage.

#### 2.1.2 DNA extraction from formalin-fixed paraffin-embedded tissues

Genomic DNA (gDNA) was isolated from serial 10 µm-thick sections cut from the FFPE tissue blocks. The extraction was performed using the DNeasy Blood & Tissue Kit (Qiagen, Hilden, Germany; Cat. No. 69504). Given the unique challenges presented by FFPE tissues, such as paraffin infiltration and formalin-induced molecular cross-linking, the standard protocol was modified to include essential pre-treatment steps to ensure maximal DNA yield and quality.

The detailed extraction protocol was as follows:

1. Deparaffinization: Tissue sections were placed into a 1.5 mL microcentrifuge tube. To remove the embedding paraffin, 1 mL of xylene was added, vortexed, and incubated for 10 min at room temperature. The sample was then centrifuged, and the supernatant was discarded. This xylene wash was repeated once.

2. Rehydration: The tissue pellet was rehydrated through a graded ethanol series. Two sequential washes with 1 mL of 100% ethanol were performed, followed by washes with 90%, 70%, and 50% ethanol to completely remove residual xylene. After the final wash, the pellet was air-dried for 10–15 min to evaporate any remaining ethanol.

3. Lysis and Digestion: The dried tissue pellet was resuspended in 180 µL of buffer ATL. To this suspension, 20 µL of Proteinase K was added. The mixture was thoroughly vortexed to ensure complete tissue immersion, then incubated overnight at 56 °C in a shaking water bath until the tissue was completely lysed.

4. Reverse Cross-linking: Following enzymatic digestion, the samples were subjected to a crucial heat treatment step to reverse the formalin-induced cross-links between nucleic acids and proteins. The lysate was incubated at 90 °C for 1 h. This step is critical for improving the yield and quality of DNA from FFPE material, making it more suitable for downstream enzymatic reactions.

5. DNA Binding and Purification: After the heat incubation, the samples were briefly centrifuged. Two hundred microliters of buffer AL and 200 µL of 100% ethanol were added to the lysate and mixed thoroughly by vortexing to create optimal conditions for DNA binding to the silica membrane. The entire mixture was then transferred to a DNeasy Mini spin column placed in a 2 mL collection tube and centrifuged at ≥6000×g for 1 min.

6. Washing: The spin column was washed twice to remove contaminants and inhibitors. First, 500 µL of buffer AW1 was added, and the column was centrifuged for 1 min at ≥6000×g. Next, 500 µL of buffer AW2 was added, and the column was centrifuged for 3 min at 20,000×g to ensure the complete removal of ethanol.

7. Elution: The DNeasy Mini spin column was transferred to a clean 1.5 mL microcentrifuge tube. The gDNA was eluted by adding 100 µL of buffer AE (10 mM Tris-Cl, 0.5 mM EDTA; pH 9.0) directly onto the center of the silica membrane. The column was incubated for 1 min at room temperature, then centrifuged for 1 min at ≥ 6000×g to collect the purified DNA.

#### 2.1.3 DNA quantification and quality assessment

The concentration and purity of the extracted double-stranded DNA (dsDNA) were determined to ensure that samples met the stringent requirements for library preparation. DNA concentration was measured using a Qubit™ 4 Fluorometer (Thermo Fisher Scientific, Waltham, MA, USA) in conjunction with the Qubit™ dsDNA HS (High Sensitivity) Assay Kit. The assay involved preparing a working solution by diluting the Qubit™ reagent 1:200 in the provided buffer, adding 1-20 µL of each DNA sample to 0.5 mL polymerase chain reaction (PCR) tubes, and adjusting the final volume to 200 µL with the working solution. Finally, the fluorescence was measured after incubation for 2 min at room temperature away from light.

In parallel, the integrity and size distribution of the gDNA were assessed via electrophoresis. An aliquot of each sample was loaded onto a 1.0% agarose gel containing ethidium bromide and run alongside a DNA ladder of known molecular weight. The presence of a high-molecular-weight band with minimal smearing was used as a qualitative indicator of high-quality, non-degraded DNA suitable for WES.

### 2.2 Whole-exome sequencing and data processing

#### 2.2.1 Library preparation and sequencing

All WES services, from library construction to sequencing, were performed by Novogene Corporation Inc. (Beijing, China). For each of the six samples, 1 µg of gDNA was used as the starting amount for library preparation. The DNA was first fragmented by sonication to a target size range of 150–200 bp.

The fragmented DNA was then used to construct sequencing libraries with the SeqCap EZ MedExome Kit (Roche, Basel, Switzerland). The library preparation workflow included sequential enzymatic steps: end-repair to create blunt-ended fragments, A-tailing to add a single adenine nucleotide to the 3’ ends, and ligation of Illumina-compatible paired-end adapters containing unique indices for each sample.

Following library construction, hybridization-based target enrichment was performed using the SeqCap EZ MedExome Library v3.0 probe set (Roche NimbleGen, Madison, WI, USA). This probe set was specifically designed to capture a comprehensive panel of medically and clinically relevant exonic regions of the human genome. The adapter-ligated libraries were hybridized to the biotinylated probes for 72 h at 47 °C to allow for specific binding of the target DNA fragments. The captured DNA-probe hybrids were then pulled down using streptavidin-conjugated magnetic beads, and non-specific DNA was removed through a series of stringent washes. The enriched libraries were subsequently amplified via PCR to generate sufficient material for sequencing.

The final, quality-controlled libraries were pooled and sequenced on an Illumina HiSeq X Ten platform, generating 125 bp paired-end reads. The sequencing was designed to achieve a target average coverage depth of approximately 200× for the cancerous tissue samples and 100× for the adjacent non-cancerous tissue samples, ensuring high-confidence variant detection across the targeted exonic regions.

#### 2.2.2 Raw read quality control and alignment

The raw sequencing data, delivered in FASTQ format, were subjected to a rigorous bioinformatics pipeline to ensure data quality and prepare for variant analysis.

Initial Quality Assessment and Trimming: The quality of the raw reads was initially assessed using FastQC (v0.11.9) (18), which provided metrics on per-base quality, GC content, sequence duplication levels, and potential adapter contamination. Based on this assessment, the raw reads were processed with Trimmomatic (v0.39) (18) to remove low-quality data and technical sequences.

Alignment and Post-Processing: The resulting high-quality, clean paired-end reads were aligned to the human reference genome assembly GRCh38/hg38 using the Burrows-Wheeler Aligner with the MEM algorithm (BWA-MEM, v0.7.17-r1188) (19). The alignment was performed using default parameters, including the -M flag, which marks shorter split hits as secondary alignments to ensure compatibility with downstream tools such as Picard and GATK. The output SAM files were converted to the compressed binary BAM format and coordinate-sorted using the sort command in SAMtools (v1.10) (20). To mitigate biases in variant calling introduced by PCR amplification during library preparation, duplicate reads were identified and marked using the Sambamba markdup tool (v0.8.2) (21). The final, analysis-ready BAM files, containing sorted reads with duplicates marked, were indexed and used for all subsequent variant detection analyses.

### 2.3 Germline variant calling and cancer susceptibility gene screening

To identify inherited genetic variants that may predispose individuals to PMMC, germline variants were called from the WES data of the three adjacent non-cancerous tissue samples. This analysis aimed to pinpoint rare, potentially pathogenic variants in known cancer predisposing genes (CPGs).

Variant Calling: Germline single-nucleotide polymorphisms (SNPs) and short InDels were identified using the SAMtools mpileup and BCFtools call pipeline. For each sample’s analysis-ready BAM file, genotype likelihoods were calculated at each position using mpileup. These likelihoods were then piped to bcftools call with the -mv options to perform variant calling using the multiallelic-aware model and to output only sites containing a variant allele.

Variant Filtering for Susceptibility Genes: The resulting raw variant calls were subjected to a rigorous, multi-stage filtering cascade designed to remove common polymorphisms and technical artifacts, thereby enriching for rare variants with a higher likelihood of being pathogenic:

1. Coverage Filter: Variants at sites with a read depth below 10× were excluded, as calls with insufficient read support are unreliable.

2. Population Frequency Filter: To isolate rare variants, any SNP or InDel with a minor allele frequency (MAF) greater than 0.001 (0.1%) in large-scale public population databases was removed. The databases used for this filtering step were the 1000 Genomes Project, the Exome Aggregation Consortium (ExAC) (22), and the NHLBI GO Exome Sequencing Project (ESP6500) (23).

3. Database Annotation Filter: Variants cataloged in the dbSNP database were filtered out to remove known common variants. However, an exception was made for variants that, despite being in dbSNP, are also annotated in the COSMIC database, as this suggests a potential role in somatic carcinogenesis.

4. Functional Consequence Filter: To focus the analysis on variants most likely to have a functional impact, variants located in intergenic regions, introns, or other non-coding regions were excluded. Additionally, synonymous mutations within coding sequences that do not alter the resulting amino acid were removed.

5. Genomic Context Filter: Variants situated within known repetitive elements or low-complexity regions of the genome were discarded, as these regions are notoriously prone to read misalignment and can lead to false-positive variant calls.

6. In Silico Pathogenicity Prediction Filter: The remaining variants were annotated with predictions of their functional impact using a suite of in silico tools. A variant was retained for final consideration only if it met stringent criteria for predicted pathogenicity: classified as “damaging” or “harmful” by at least one of the following algorithms—SIFT, PolyPhen-2 HumVar, PolyPhen-2 HumDiv, or MutationTaster—or classified as “possibly damaging” by two or more of these tools.

### 2.4 Somatic variant calling

Somatic mutations—genetic alterations unique to the tumor cells—were identified by comparing the WES data from each tumor sample against its matched adjacent non-cancerous tissue sample. This paired analysis is essential for distinguishing true somatic events from germline variants. For enhanced accuracy and sensitivity, somatic single-nucleotide variants (SNVs) and InDels were called using the GATK4 MuTect2 (v4.2.0.0) (24) pipeline, following the GATK Best Practices for somatic short variant discovery.

For each of the three tumor-normal pairs, MuTect2 was run in paired mode, providing both the tumor and normal BAM files as input. This allows the algorithm to construct a model of the normal sample’s germline variation and identify variants present only in the tumor with high confidence. The raw VCF output from MuTect2 was then processed with GATK’s FilterMutectCalls tool, which applies a series of learned filters to flag and remove common sequencing artifacts and low-confidence calls. Only variants that passed all filters (i.e., had “PASS” in the FILTER column of the VCF file) were retained. Finally, these high-confidence somatic variants were annotated to determine their location and to predict their effect on protein function, and only those occurring within coding regions were carried forward for downstream analyses.

### 2.5 Mutational signature and spectrum analysis

#### 2.5.1 *De novo* mutational signature extraction

To investigate the underlying mutational processes active in PMMC, the spectrum of somatic SNVs was analyzed to extract mutational signatures. The mutational catalog for each of the three tumor samples was constructed by classifying each high-confidence somatic SNV based on its six possible substitution types (C>A, C>G, C>T, T>A, T>C, T>G) and the immediate 5’ and 3’ flanking nucleotide bases. This classification results in a profile of 96 distinct mutation types per tumor.

*De novo* extraction of mutational signatures from these 96-channel profiles was performed using the SigProfilerExtractor Python package (v1.1.21) (25). This tool utilizes an optimized, iterative Nonnegative Matrix Factorization (NMF) algorithm to deconvolve the mutation catalogs into a set of mutational signatures and their corresponding activities (exposures) in each sample. The analysis was executed with parameters optimized for WES data. Specifically, the number of signatures to extract was explored in the range of 1 to 10 (minimum_signatures=1, maximum_signatures=10), with 100 NMF replicates per run (nmf_replicates=100). The optimal number of signatures was automatically selected by the software to balance maximizing solution stability and minimizing reconstruction error. To account for the differential trinucleotide frequencies in targeted regions compared to the whole genome, the exome parameter was set to True.

The profiles of the extracted *de novo* signatures were subsequently compared against the reference set of known signatures from the COSMIC (Catalogue of Somatic Mutations in Cancer) v3.0 database (released May 2019) (https://cancer.sanger.ac.uk/cosmic), which contains 67 established single-base substitution (SBS) signatures. A cosine similarity score > 0.80 was used to confidently associate an extracted signature with a known COSMIC signature. Any *de novo* signature that did not meet this similarity threshold with any known signature was considered potentially novel to PMMC.

#### 2.5.2 Justification for the use of *de novo* signature detection for WES data

While signature refitting—the process of estimating the contributions of a predefined set of known signatures to a sample’s mutation catalog—is a widely used and powerful method, a *de novo* discovery approach was deliberately chosen for this study. This decision was based on a strong scientific rationale centered on the nature of the disease under investigation.

PMMC is a rare malignancy, and as such, its molecular etiology and the full spectrum of mutational processes that drive its development are not well characterized. The comprehensive COSMIC reference signature database has been predominantly built from large-scale analyses of more common cancer types. Consequently, relying solely on a refitting approach would presuppose that the mutational processes active in PMMC are a subset of those already described. This carries a substantial risk of either misattributing the observed mutational patterns to ill-fitting combinations of known signatures or, more critically, failing to identify potentially novel, PMMC-specific signatures that are absent from the reference catalog.

### 2.6 Identification of driver genes and significantly mutated genes

#### 2.6.1 Driver gene screening

To identify mutations in genes with a known role in cancer, the list of all genes harboring high-confidence somatic mutations (both SNVs and InDels) was cross-referenced against a comprehensive, aggregated database of cancer driver genes. This database was compiled from four authoritative sources to maximize coverage: the Cancer Gene Census (CGC, v94) (26), the list of 125 mut-driver genes published by Vogelstein et al. (27), a pan-cancer study identifying 127 significantly mutated genes (28), and a composite list of 435 driver genes (29) identified by multiple computational methods.

#### 2.6.2 Significantly mutated genes and pathway enrichment analysis

To statistically identify genes that were mutated more frequently than expected by chance within the PMMC cohort, we performed an SMG analysis. This was conducted using the smg module of the MuSiC (Mutational Significance in Cancer) suite (v2.0) (30). This tool integrates information on both SNVs and InDels and applies a convolution test (CT) to assess whether the observed mutation frequency of a given gene is significantly higher than the background mutation rate (BMR), which is calculated based on gene length, sequence composition, and cohort-wide mutation rates. Genes with a False Discovery Rate (FDR) adjusted p-value, or Q-value, < 0.2 were classified as SMGs in PMMC.

To elucidate the biological pathways and processes predominantly affected by these high-frequency mutations, the identified SMGs were subjected to pathway enrichment analysis using the PathScan software (31). The analysis tested for the over-representation of the SMG list in canonical pathways curated in the Kyoto Encyclopedia of Genes and Genomes (KEGG) database. P-values from the enrichment analysis were adjusted for multiple testing using the Benjamini-Hochberg procedure. Pathways with an adjusted P-value < 0.05 were considered significantly enriched.

### 2.7 Copy number variation analysis

Somatic CNVs, representing gains (amplifications) and losses (deletions) of genomic segments in the tumor cells, were detected from the WES data using Control-FREEC (v11.6) (32). This tool analyzes read counts in predefined genomic windows to infer copy-number status. For each tumor sample, the matched normal sample was used as a control to establish a baseline diploid copy-number, enabling the specific detection of somatic events.

The software was configured with parameters optimized for targeted sequencing data, like WES, which is characterized by non-uniform coverage. Key parameters included: degree = 1 (for control-read-count-based normalization), forceGCcontentNormalization = 1 (to correct for GC-content bias), minCNAlength = 3 (requiring a CNA to span at least three consecutive windows), minimalSubclonePresence = 30 (to detect subclones present in at least 30% of cells), noisyData = TRUE (to account for non-uniform capture efficiency), and minMappabilityPerWindow = 0.85 (to exclude windows with low mappability).

### 2.8 Comparative analysis with public cancer datasets

To place the genomic alterations found in PMMC into a broader context of skin malignancies, the mutational landscape of PMMC was compared with that of SKCM, a well-characterized cancer type.

Data Acquisition: Publicly available somatic mutation data for SKCM were acquired from two major cancer genomics databases. First, data for 1,366 SKCM cases were extracted from the COSMIC database (v94) (https://cancer.sanger.ac.uk/cosmic) using the search terms “skin” for the primary site and “malignant melanoma” for the disease type. Secondly, Masked Somatic Mutation (SNV) data for 467 patients with SKCM and 289 patients with cervical cancer (CC) were downloaded from The Cancer Genome Atlas (TCGA) portal (https://portal.gdc.cancer.gov/).

Intersection Analysis: A list of gene mutation frequency was generated from the TCGA SKCM cohort. To identify somatic mutations potentially unique to PMMC, the list of SMGs identified in this study was intersected with two lists derived from the SKCM data (1): the top 300 most frequently mutated genes in the TCGA cohort, and (2) genes mutated in 10 or more samples in the COSMIC SKCM cohort. Genes present in the PMMC SMG list but absent from both SKCM lists were highlighted as candidate genes specific to the pathobiology of PMMC.

CNV Comparison: For a comparative analysis of large-scale genomic alterations, Masked Copy Number Segment data for 472 TCGA SKCM tumor samples were also downloaded from the TCGA portal (data accessed on May 31, 2021) to compare CNV profiles between PMMC and SKCM.

### 2.9 Cell culture

HMV-II human melanoma cells were purchased from Sigma-Aldrich (92042701, USA). HMV-II cells were removed from liquid nitrogen and quickly placed in a 37 °C water bath. The cryovial was gently shaken to thaw. After thawing, the cells were transferred to a centrifuge tube containing 5 mL of culture medium and centrifuged at 1000 rpm for 5 min at room temperature. The supernatant was discarded, and the cells were resuspended in RPMI 1640 Medium (Gibco, USA) supplemented with fetal bovine serum, plated onto a culture dish, and gently pipetted to mix. The cells were incubated at 37 °C in a humidified atmosphere of 5% CO2. Cells were passaged when the cell density reached 80% and carefully handled to prevent contamination and authenticated by short tandem repeat sequence analysis to ensure quality.

### 2.10 Establishment of HMV-II cell lines with knockout of P53, AKT3, SMYD4, and ATAD3A

HMV-II cells were seeded at a density of 5 × 10^5^ cells per well in a 6-well plate. Next, 15 nM siRNA-1 or siRNA-2 and siRNA NC (GenePharma, Shanghai, China) were transfected into HMV-II cells using Lipofectamine 3000 (Thermo, New York, USA). The sequence of siRNA was: P53-siRNA-1: AGACCUAUGGAAACUACUU; P53-siRNA-2: CCAGAUGAAGCUCCCAGAA; AKT3-siRNA-1: UCAUCUUUCUCCUUCAUUA; AKT3-siRNA-2: AGAGGUGUUAGAAGAUAAU; SMYD4-siRNA-1: AGACUCCAUCUCAAAGAAA; SMYD4-siRNA-2: AAAGGUUACUUGGUGGGAA; ATAD3A-siRNA-1: ACUUCUCAAUGAGGAGAAU; ATAD3A-siRNA-2: AGGACAAAUGGAGCAACUU; siRNA-NC: UUCUCCGAACGUGUCACGUTT.

### 2.11 Establishment of HMV-II cell line overexpressing SIRPB1, AHNAK2, and XKR6

To overexpress SIRPB1, AHNAK2, and XKR6, plasmids containing the coding sequences of these three genes or empty vectors were transfected into HMV-II cells (**Supplementary Table 2**). See 2.11 for the transfection procedure.

### 2.12 Cell Counting Kit-8 assay

After washing with phosphate-buffered saline (PBS), cells were trypsinized and resuspended in complete culture medium. Cells were seeded in 96-well plates (5,000 cells per well). CCK-8 reagent (Promega, USA) was added to each well after 1, 2, 3, and 4 days. After incubation at 37 °C for 1 h, the optical density (OD) at 450 nm was measured. Experiments were repeated three times.

### 2.13 Transwell assay

Transwell assays were performed using Corning Transwell chambers (Corning, USA) featuring 8-μm membrane pores. First, 15 μL of diluted Matrigel (diluted at a ratio of 1:8) was added to the upper chamber, followed by seeding 5 × 10^5^ HMV-II cells. After 24 h of incubation, the upper chamber was removed, and the excess liquid in it was discarded. The cells attached to the lower surface of the upper chamber were fixed in methanol for 30 min at room temperature and then stained with 0.1% crystal violet for 10–20 min at room temperature. The cell images were captured using a microscope. This experiment was repeated three times.

### 2.14 Real-time fluorescence quantitative PCR

HMV-II cells were lysed using TRIzol reagent (Invitrogen, New York, USA) to extract total RNA. qRT-PCR analysis was carried out with the HiScript II One Step qRT-PCR SYBR Green kit (Vazyme, Jiangsu, China) on a Bio-Rad CFX96 PCR system (Bio-Rad, Hercules, California, USA). Primers were designed and synthesized by RuiBiotech (Beijing, China). Using GAPDH as the internal reference, the expression levels of target genes were calculated via the 2^−ΔΔCt^ method. Primer sequences are presented in **Supplementary Table 3**. The experiment was repeated three times.

### 2.15 Western blotting and co-immunoprecipitation

We extracted total proteins from cells using a lysis buffer (AR0105, Boster, China). Subsequently, the proteins were separated by 10% sodium dodecyl sulfate-polyacrylamide gel electrophoresis and then transferred onto a polyvinylidene fluoride (PVDF) membrane. Prior to adding specific primary antibodies, the membrane was blocked with 5% nonfat milk at room temperature for 1.5 h. After that, the membrane was incubated overnight at 4 °C with the following primary antibodies: anti-P53 (345567, 1:1000) and anti-ATAD3A (124203, 1:1000) from Zenbio, and anti-AKT3 (21641, 1:1000), anti-SMYD4 (17594, 1:1000), anti-NF-κB (66535, 1:1000), anti-p-NF-κB (82335, 1:2000), anti-AKT (10176, 1:2000), and anti-p-AKT (66444, 1:2000) from Proteintech. To detect the protein bands, the membrane was incubated with a horseradish peroxidase-conjugated secondary antibody and an enhanced chemiluminescence reagent (AR1191S, Boster, China). Finally, band density was quantified using ImageJ software and normalized to the loading control.

For Co-IP analysis, the treated cell lysates were incubated with target antibodies overnight at 4 °C. Then, immunoprecipitation was carried out using Protein A+G agarose (P2012, Beyotime, China) for 4 h at 4 °C. Subsequently, the samples were subjected to western blot analysis to detect co-precipitated proteins. The total cell lysates were also analyzed by western blotting as input controls. Finally, the protein interactions were quantitatively analyzed using ImageJ software.

### 2.16 Immunohistochemical staining

We stained paraffin sections using an immunohistochemical staining kit (Elabscience, China, E-IR-R215). Tissue sections were first dewaxed, hydrated, subjected to antigen retrieval, inactivated for endogenous enzymes, and blocked with serum. Anti-SIRPB1 (11811, 1:200, Proteintech), anti-AHNAK2 (17682, 1:200, Proteintech), and anti-XKR6 (23968, 1:200, Proteintech) were then applied to the sections and incubated at 37 °C for 2 h. After three washes with PBS, secondary antibodies (Elabscience, China, E-IR-R215B) were applied, incubated at 37 °C for 30 min, and then washed thrice with PBS. Finally, DAB staining solution was applied to the sections; a brownish-yellow color indicated a positive result. Pathological images were quantified and analyzed using ImageJ.

### 2.17 Statistical analysis

All data in this study were based on at least three biological replicates, and all statistical analyses were conducted in R (version 4.4.1). The Mann-Whitney U test was used for intergroup comparisons. PathScan was used for pathway enrichment analysis to analyze KEGG-related signaling pathways associated with high-frequency mutation genes; the screening condition was P.adjust < 0.05. SKCM in the TCGA database uses the Chi-squared (χ2) test to analyze differences between the tumor and non-tumor groups CNV. All P-values were calculated using two-tailed tests, and a P-value < 0.05 was considered statistically significant.

## 3 Results

### 3.1 Overview of genomic alterations in primary malignant melanoma in the cervix

The surgically resected tissues from three patients with PMMC underwent WES (tumor sample: 200×depth, normal tissue: 100×depth) (**Supplementary Table 4**). We used the muTect software (33) to search for Somatic SNV sites and showed that MM01, MM02, and MM03 have Somatic SNVs of 282, 871, and 381, respectively. The vast majority of SNVs are distributed in the exon coding region (CDS) and intronic. The SNVs in the CDS are mainly synonymous mutations and missense mutations (**Table 1**).

**Table 1:** The number of somatic single-nucleotide variations (SNVs) in different regions of the PMMC genome.

Sample	MM01	MM02	MM03
CDS	91	332	160
synonymous_SNP	25	106	96
missense_SNP	63	213	63
stopgain	2	13	0
stoploss	0	0	0
unknown	1	0	1
intronic	102	368	143
UTR3	10	25	11
UTR5	9	12	13
splicing	2	8	2
ncRNA_exonic	12	21	8
ncRNA_intronic	16	26	8
ncRNA_UTR3	0	0	0
ncRNA_UTR5	0	0	0
ncRNA_splicing	0	0	1
upstream	4	7	4
downstream	5	4	4
intergenic	31	67	27
Others	0	1	0
Total	282	871	381

InDels in the coding region or at a splice site may alter protein translation. Frameshift mutation occur when the length of an inserted or deleted base string is a non-integer multiple of 3, causing the entire reading frame to shift. Compared with non-frameshift mutations, frameshift mutation exert a greater impact on gene function, and are also subjected to stronger screening pressure (34). Using Strelka to detect Somatic InDel information, this study showed that MM01, MM02, and MM03 have 16, 30, and 99, Somatic InDels respectively. The vast majority of InDel occurs in the CDS and intronic regions, among which exons, the coding regions are mainly frameshift deletion, frameshift insertion, and non-frameshift deletion (**Table 2**).

**Table 2:** Somatic cell insertion-deletion (InDel) detection in different regions of the PMMC genome.

Sample	MM01	MM02	MM03
CDS	7	12	25
frameshift_deletion	3	4	7
frameshift_insertion	2	3	0
nonframeshift_deletion	2	4	9
nonframeshift_insertion	0	1	9
stopgain	0	0	0
stoploss	0	0	0
unknown	0	0	0
intronic	7	14	35
UTR3	0	1	19
UTR5	0	2	12
splicing	0	0	0
ncRNA_exonic	0	0	2
ncRNA_intronic	0	0	5
ncRNA_UTR3	0	0	0
ncRNA_UTR5	0	0	0
ncRNA_splicing	0	0	0
upstream	1	0	1
downstream	0	0	0
intergenic	1	1	0
Others	0	0	0
Total	16	30	99

### 3.2 Primary malignant melanoma in the cervix susceptibility gene screening

**Figure 1:**
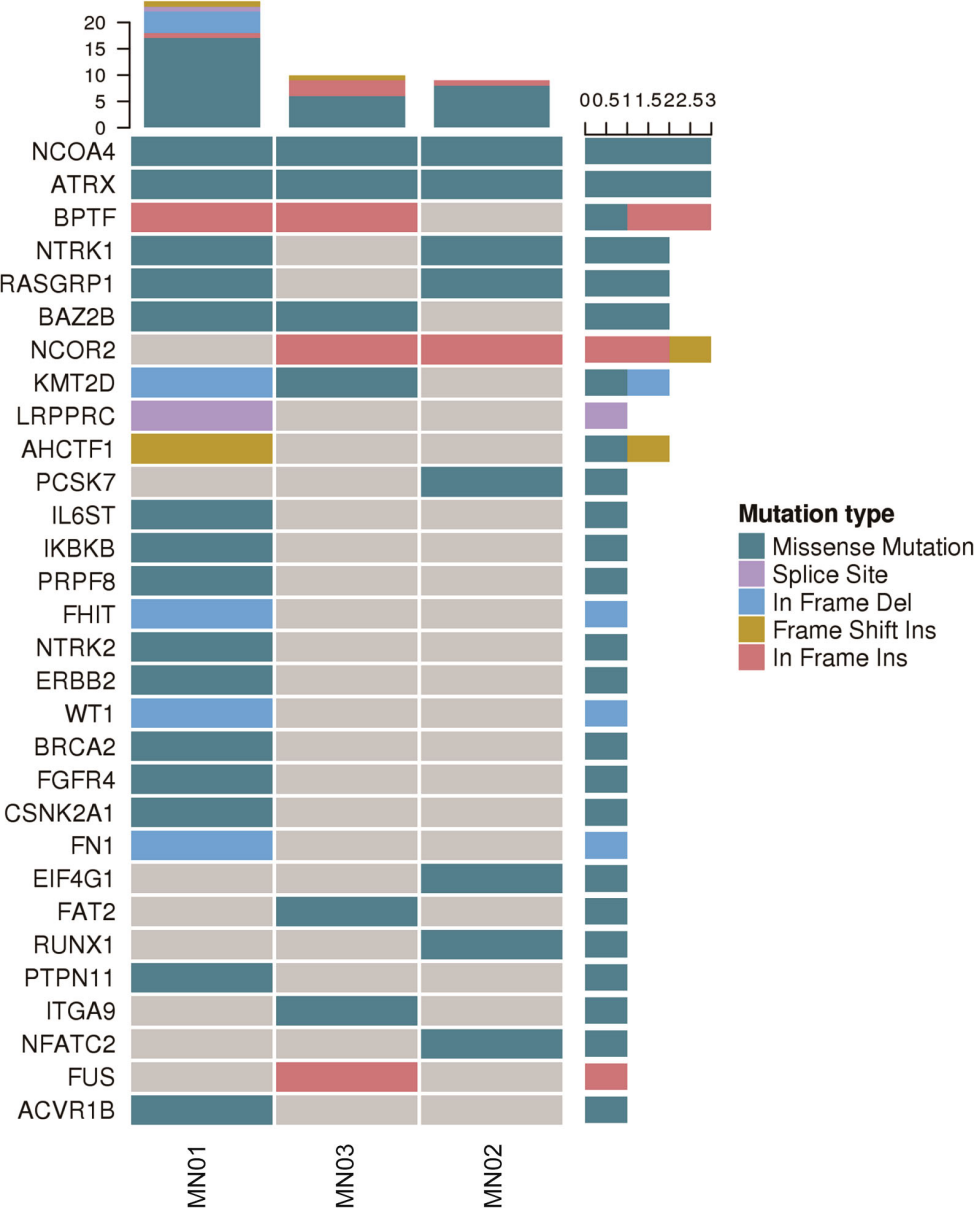
Landscape map of cancer predisposing genes in PMMC patients. Mutation types include missense mutation, splice site, in-frame del, frameshift ins, and in-frame ins.

Samtools software was used to detect Germline Mutations (SNPs, InDels) in the patient’s normal tissues, and then the detected mutant genes were screened for possible cancer-predisposing genes. Finally, using the Cancer Gene Census (CGC) database (http://cancer.sanger.ac.uk/cancergenome/projects/census/), the FACD (Familial Cancer Database, http://www.familialcancerdatabase.nl/) database, intoGen (Rubio-Perez C, 2015) database, the above screening results were annotated. In this study, the susceptibility genes in the adjacent tissues of PMMC were mainly *NCOA4*, *ATRX*, *BPTF*, *NTRK1*, *RASGRP1*, *BAZ2B*, *NCOR2*, *KMT2D*, and the main mutation types were Missense Mutation and In Frame Ins (**Figure 1**, **Supplementary Table 5**).

### 3.3 Mutation spectrum and signature of primary malignant melanoma in the cervix

**Figure 2:**
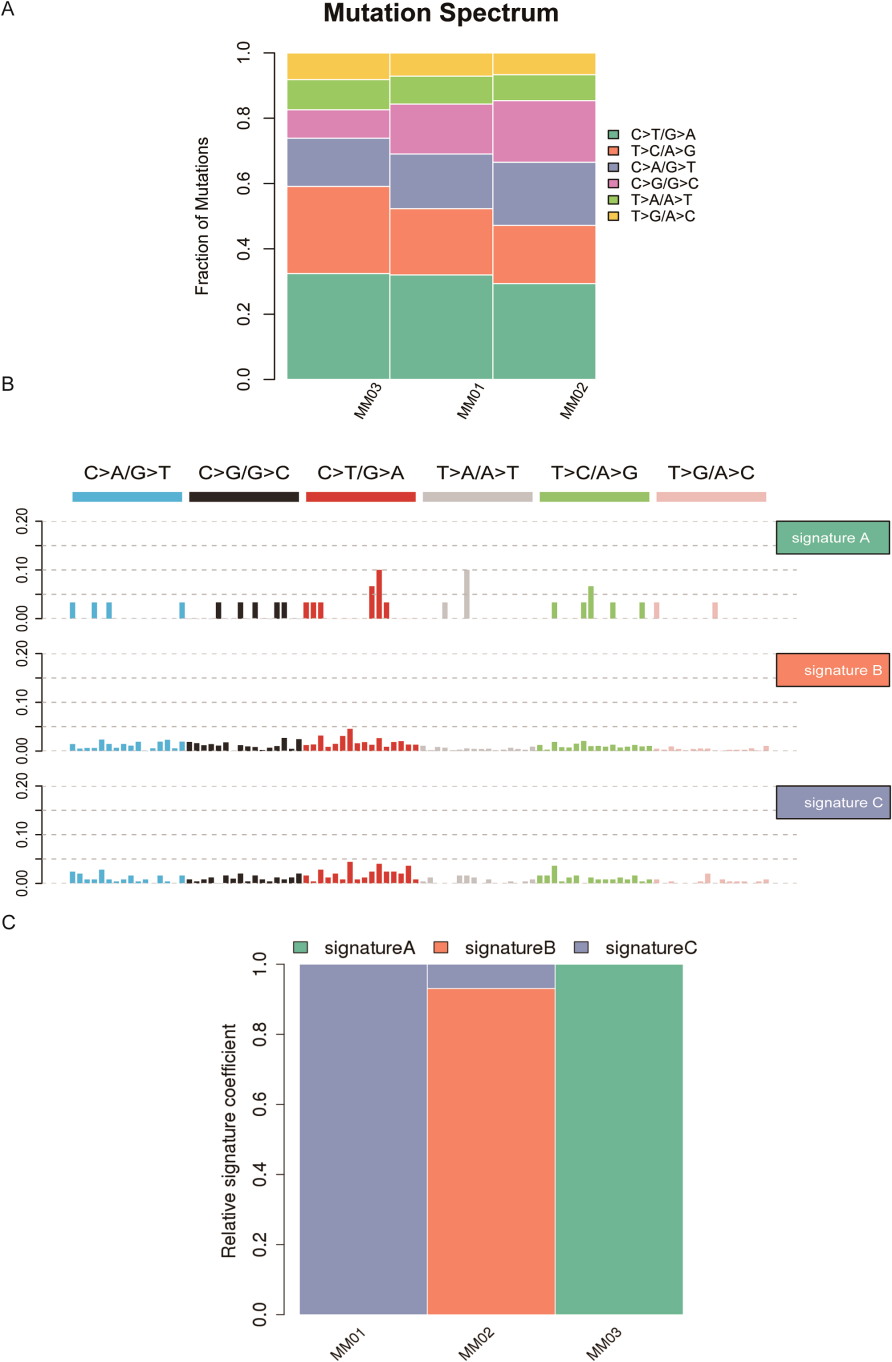
Analysis of mutation spectrum and mutation characteristics in patients with PMMC. **(A)** Histogram of Abrupt Spectrum, there are six types of point mutations: C>A/G>T, C>G/G>C, C>T/G>A, T>A/A>T, T>C/A>G, T> G/A>C. The abscissa of the mutation spectrum histogram is the sample name, and the ordinate is the proportion of each mutation type in the sample. Different colors represent different types of single-nucleotide variation (SNV) mutation. **(B)** Mutation feature display map. The NMF algorithm was used to cluster 96 types of mutations in tumor samples, and the mutation characteristics were obtained. Each mutation characteristic characterizes the tumor mutation process. **(C)** Proportion of each mutation feature in different samples. The abscissa shows the sample, and the ordinate shows the ratio of each mutation feature.

In our PMMC study, the most common mutation type was C>T/G>A, followed by T>C/A>G, C>A/G>T, C>G/G>C, T>A/A>T, and T>G/A>C (**Figure 2A**). Based on the frequency of 96 mutation types in PMMC samples, we performed NMF clustering and identified three mutation features (signature A, signature B, and signature C) (**Figure 2B**). MM01 and MM03 corresponded to signatures C and A, respectively, and MM02 corresponded to signature B (**Figure 2C**). After that, unsupervised hierarchical clustering of cosine similarity values further identified signature A (cosine similarity = 0.77). Moreover, signature C (cosine similarity = 0.75) showed a similarity with SBS5, and signature B (cosine similarity = 0.79) with SBS40 (**Supplementary Figure 1**). SBS5 showed similarity with the signature of a clock, with number of mutations correlating with aging, and SBS40 was also found to be associated with the age of cancer patients (**Supplementary Table 6**).

### 3.4 Analysis of significantly mutated genes of primary malignant melanoma in the cervix landscape and gene enrichment

**Figure 3:**
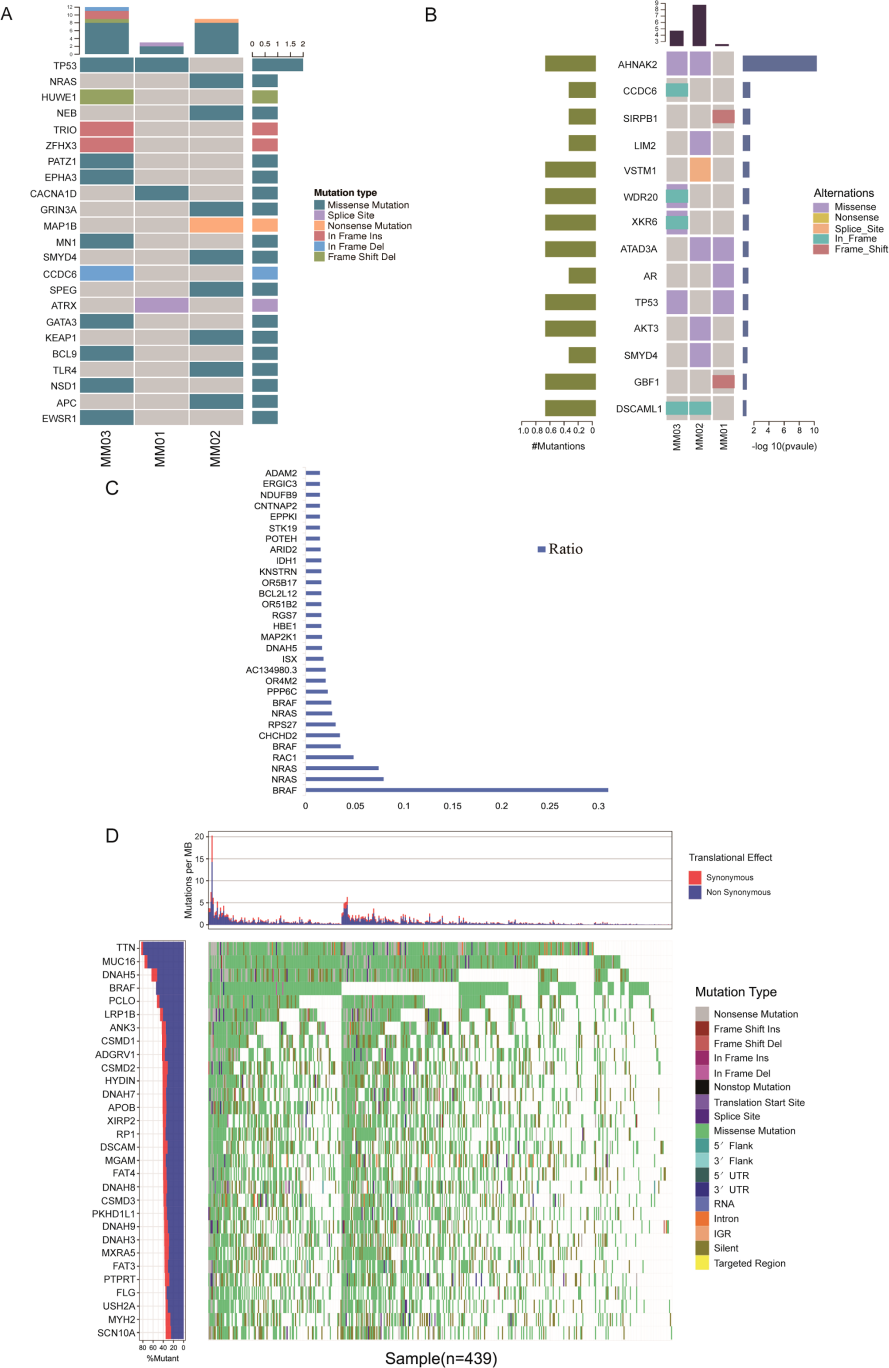
Screening of driver genes in patients with PMMC and analysis of high-frequency tumor mutations. **(A)** Landscape map of known driver genes. The abscissa is the sample, the ordinate is the gene, the top presents the number of mutations in each sample for each gene, and the right presents the number of mutations in each gene in these samples. **(B)** High-frequency mutation heat map of cancer somatic cells. The top bar graph shows the mutation rate (mutations/Mb) of each sample. The heat map below shows the high-frequency mutation genes and mutation types. The abscissa is the sample name, and the ordinate is the high-frequency mutation gene. The different colors in the map represent different types of mutation. The bar graph on the left side of the heat map shows the proportion of samples with mutations in each gene, and the right side shows the log10 (p-value) of the highly mutated genes. **(C)** Main map of melanoma gene mutation rate in the COSMIC database. **(D)** The Cancer Genome Atlas (TCGA) database melanoma SNP waterfall chart.

First, we compared the somatic mutations of the tumor sample with the known driver genes in the database and screened out the known driver genes in the tumor sample. It was found that the driver gene shared by MM01 and MM03 was *TP53*. The driver genes of MM01 are *CACNA1D* and *ATRX*, MM03 are *PATZ1*, *EPHA3*, *MN1*, *GATA3*, *BCL9*, *NSD1*, and *EWSR1*. The driver genes of MM02 are different from MM01 and MM03, and are *NRAS*, *NEB*, *GRIN3A*, *SMYD4*, *SPEG*, *KEAP1*, *TLR1*, and *APC* (**Figure 3A**). Driver gene mutations in PMMC tumor samples are similar to those in para-cancerous tissues, and remain dominated by Missense and In Frame Ins (**Supplementary Table 7**).

After comprehensively considering the somatic SNV and InDel mutations, the high-frequency mutation genes were screened out as *AHNAK2*, *SIRPB1*, *XKR6*, *ATAD3A*, *TP53*, *AKT3*, *SMYD4*, *GBF1*, and *DSCAML1* (**Figure 3B**). The main types of mutations were missense and in-frame (**Supplementary Table 8**).

Further analysis of 1366 SKCM patients in the COSMIC database, the top 30 genes were *BRAF*, *NRAS*, *RAC1*, *CHCHD2*, *RPS27*, *PPP6C*, *OR4M2*, *AC134980.3*, *ISX*, *DNAH5*, *MAP2K1*, *HBE1*, *RGS7*, *OR51B2*, *BCL2L12*, *OR5B17*, *KNSTRN*, *IDH1*, *ARID2*, *POTEH*, *STK19*, *EPPK1*, *CNTNAP2*, *NDUFB9*, *ERGIC3*, and *ADAM2* (**Figure 3C**). At the same time, analysis of 467 SNP data of patients with SKCM in the TCGA database revealed the first 30 genes as *TTN*, *MUC16*, *DNAH5*, *BRAF*, *PCLO*, *LRP1B*, *ADGRV1*, *ANK3*, *CSMD1*, *CSMD2*, *HYDIN*, *APOB*, *DNAH7*, *XIRP2*, *MGAM*, *RP1*, *DSCAM*, *FAT4*, *DNAH8*, *CSMD3*, *PKHD1L1*, *DNAH9*, *DNAH3*, *MXRA5*, *FAT3*, *PTPRT*, *MYH2*, *FLG*, *SCN10A*, and *USH2A* (**Figure 3D**). At the same moment, when we analyzed the SNP data of 289 patients with CC in the TCGA database, we found that the first 30 genes were *TTN, PIK3CA, KMT2C, MUC16, KMT2D, FLG, EP300, DMD, SYNE1, FBXW7, LRP1B, RYR2, DST, USH2A, ADGRV1, HUWE1, MUC17, LRP2, SYNE2, NAV3, NEB, PCLO, CREBBP, DNAH2, FAT1, MDN1, MUC5B, CSMD1, PRKDC*, and *PTEN* (**Supplementary Figure 2**).

**Figure 4:**
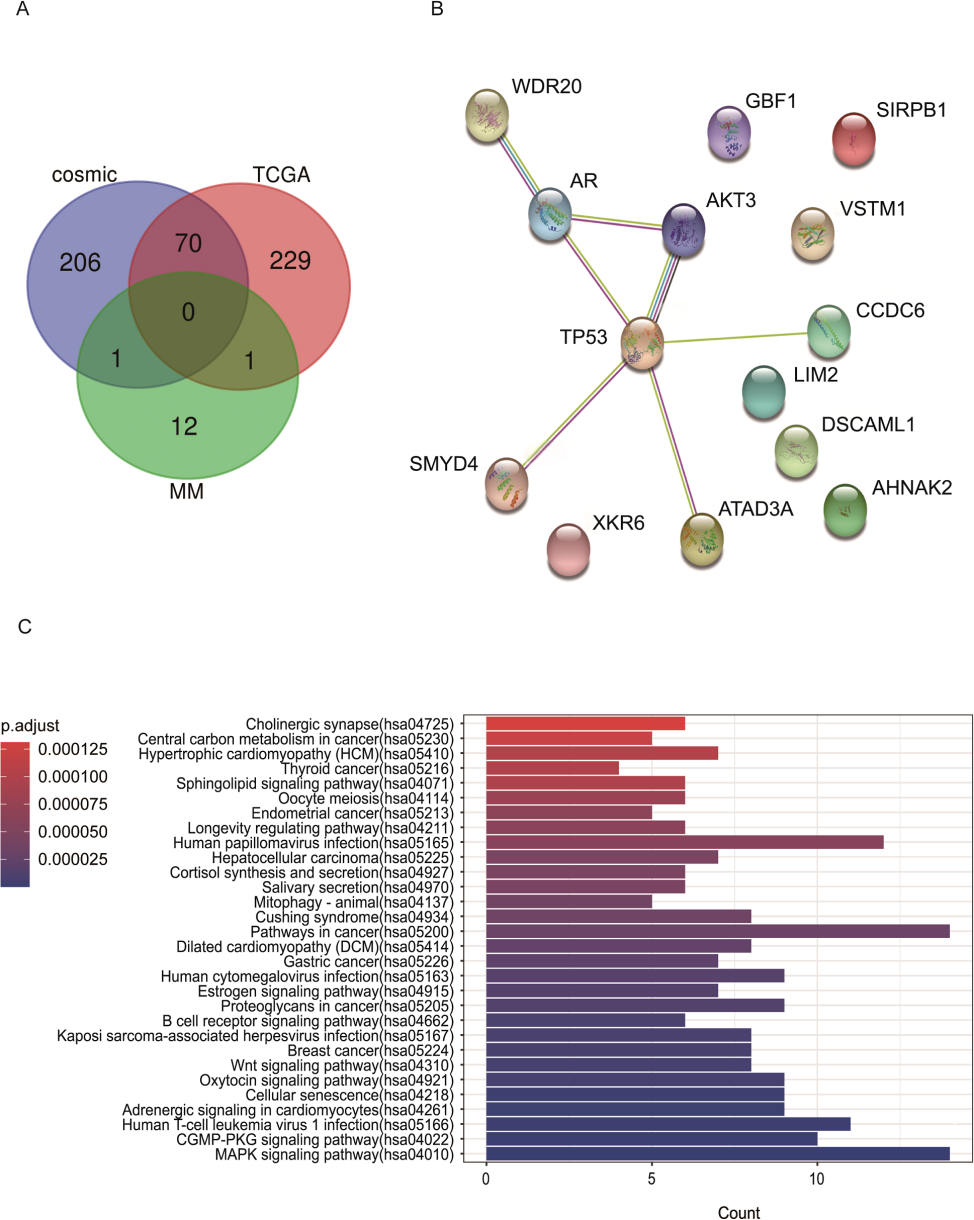
**(A)** The COMIC, TCGA database, and SMG Venn diagram of patients with PMMC. **(B)** Protein-protein interaction network of significantly mutated genes (SMG) for patients with PMMC, coefficients of |r| >0.4 and P < 0.05. **(C)** Kyoto Encyclopedia of Genes and Genomes (KEGG) enrichment analysis of SMG in patients with PMMC. The color of the bar indicates the significance of KEGG enrichment (P.adjust).

The high-frequency mutation genes in PMMC were compared with genes mutated ≥10 times in COSMIC SKCM and with the top 300 most frequently mutated genes of TCGA SKCM. The gene shared between COSMIC, CC, and PMMC was *TP53*; the gene shared by TCGA and PMMC was *AHNAK2*; and the gene shared by CC and PMMC was *DSCAML1* (**Supplementary Figure 3**). In addition, *CCDC6*, *SIRPB1*, *LIM2*, *VSTM1*, *WDR20*, *XKR6*, *ATAD3A*, *AR*, *AKT3*, *SMYD4*, *GBF1*, and *DSCAML1* were identified as high-frequency genes in PMMC, while their mutation frequencies were lower in COSMIC and TCGA (**Figure 4A**).

Finally, using the STRING website, WDR20, AR, TP53, AKT3, CCDC6, SMYD4, ATAD3A can form a protein-protein interaction network (**Figure 4B**); PathScan software analysis of high-frequency mutation gene: metabolic pathways mainly focus on mitogen-activated protein kinase (MAPK) signaling pathway, cGMP-PKG signaling pathway, Human T-cell leukemia virus 1 infection, adrenergic signaling in cardiomyocytes, cellular senescence, oxytocin signaling pathway, Wnt signaling pathway, breast cancer, Kaposi sarcoma-associated herpesvirus infection, B cell receptor signaling pathway, proteoglycans in cancer, estrogen signaling pathway, Human cytomegalovirus infection, gastric cancer, dilated cardiomyopathy, pathways in cancer, Cushing syndrome, mitophagy animal, salivary secretion, cortisol synthesis and secretion, hepatocellular carcinoma, Human papillomavirus infection, longevity regulating pathway, endometrial cancer, oocyte meiosis, sphingolipid signaling pathway, thyroid cancer, hypertrophic cardiomyopathy, central carbon metabolism in cancer, and cholinergic synapse (**Figure 4C**, **Supplementary Table 9**).

### 3.5 P53, AKT3, SMYD4, and ATAD3A play an oncogenic role in the HMV-II cell line

**Figure 5:**
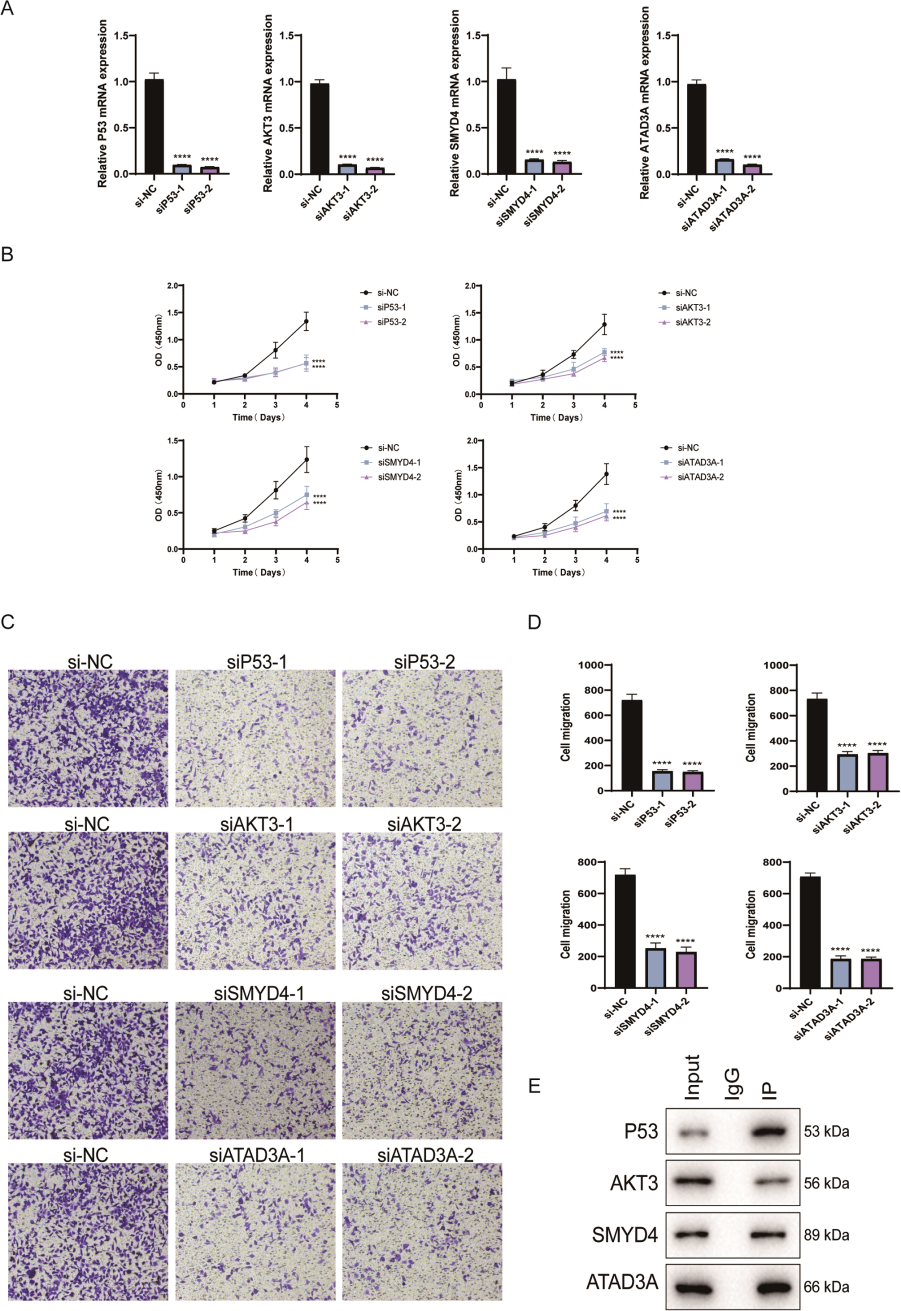
P53, AKT3, SMYD4, and ATAD3A affect the biological behavior of HMV-II. **(A)** SiRNAs were used to knock down the mRNA expression of *P53*, *AKT3*, *SMYD4*, and *ATAD3A* in HMV-II. **(B)** Effects of knockout of *P53*, *AKT3*, *SMYD4*, and *ATAD3A* on the proliferation of HMV-II cells. **(C, D)** Effects of *P53*, *AKT3*, *SMYD4*, and *ATAD3A* knockout on HMV-II cell invasion. **(E)** Co-immunoprecipitation validation of the interaction between P53, AKT3, SMYD4, and ATAD3A. *, p < 0.05; **, p < 0.01; ***, p <0.001; ****, p < 0.0001.

We selected P53-related *AKT3*, *SMYD4*, and *ATAD3A* as candidate genes. We then used siRNA to knock down *P53*, *AKT3*, *SMYD4*, and *ATAD3A* in HMV-II cells. qPCR results showed that both siRNA-1 and siRNA-2 reduced the mRNA expression of *P53* (P < 0.0001), *AKT3* (P < 0.0001), *SMYD4* (P < 0.0001), and *ATAD3A* (P < 0.0001) compared with the siRNA NC group (**Figure 5A**). We then further evaluated the effects of knockdown of these four candidate genes on cell proliferation and invasion. We found that knockdown of *P53* (P < 0.0001), *AKT3* (P < 0.0001), *SMYD4* (P < 0.0001), and *ATAD3A* (P < 0.0001) significantly reduced cell proliferation (**Figure 5B**) and invasion (**Figures 5C, D**) compared to their levels in the siRNA NC group. Based on this, we further explored the interactions between AKT3, SMYD4, ATAD3A, and P53. Co-IP experiments confirmed direct interactions between P53 and AKT3, SMYD4, and ATAD3A (**Figure 5E**).

### 3.6 The copy number variation burden of primary malignant melanoma in the cervix and skin melanoma

**Figure 6:**
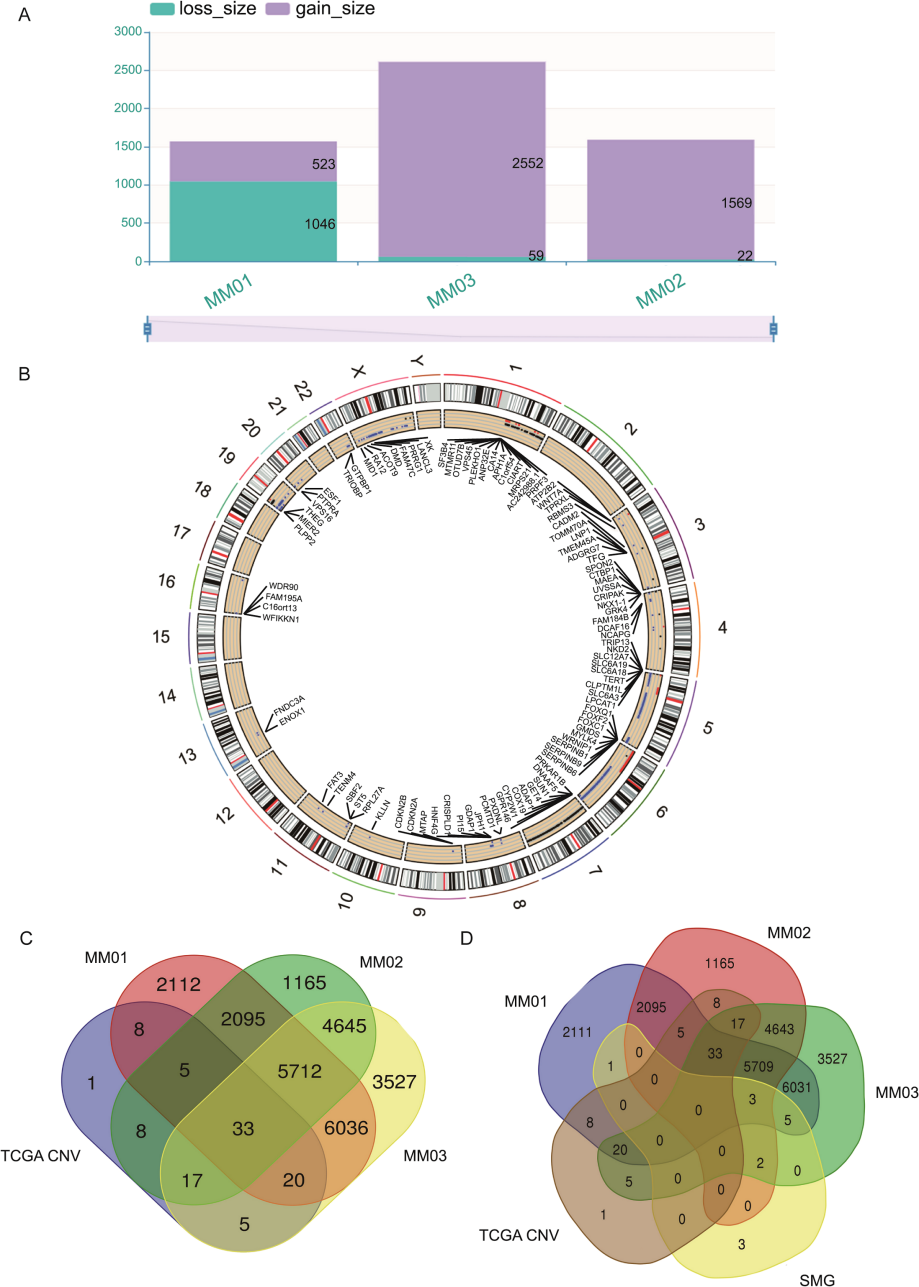
Copy number variation (CNV) analysis of patients with PMMC. **(A)** Distribution of somatic CNV. The ordinate represents the total length of the copy number (Mb), and the abscissa represents different samples. **(B)** The Cancer Genome Atlas database melanoma CNV analysis. Red indicates an increase in the copy number, blue indicates a missing copy number, and black indicates a normal copy number. **(C)** Venn map of CNV in patients with PMMC and CNV in The Cancer Genome Atlas (TCGA) data of melanoma patients. **(D)** Venn map of significantly mutated genes (SMG) and CNV in patients with PMMC and CNV in the TCGA data of melanoma patients.

Somatic CNVs in PMMC samples were assessed, revealing 114 gains and 40 losses in MM01, 93 gains and 10 losses in MM02, and 194 gains and 16 losses in MM03 (**Figure 6A**, **Supplementary Table 10**). The CNV burden in the three PMMC samples was further visualized using the Circos tool (**Supplementary Figure 4**, **Supplementary Figure 5**, **Supplementary Figure 6**). In TCGA SKCM (472 tumor samples and 467 non-tumor samples), 97 genes showed differential CNVs between tumor and normal groups (**Figure 6B**). When TCGA SKCM CNVs were compared with those from the three patients with PMMC (**Figure 6C**), 33 CNV genes were shared, and 5,712 genes exhibited CNVs only in 3 PMMCs. Intersecting these results with previously identified SMG showed that *SIRPB1*, *AHNAK2*, and *XKR6* carried both CNVs and high-frequency mutations in all three PMMC samples (**Figure 6D**).

### 3.7 Effects of copy number variation on *SIRPB1*, *AHNAK2* and *XKR6* gene expression and cellular pathway functionality

**Figure 7:**
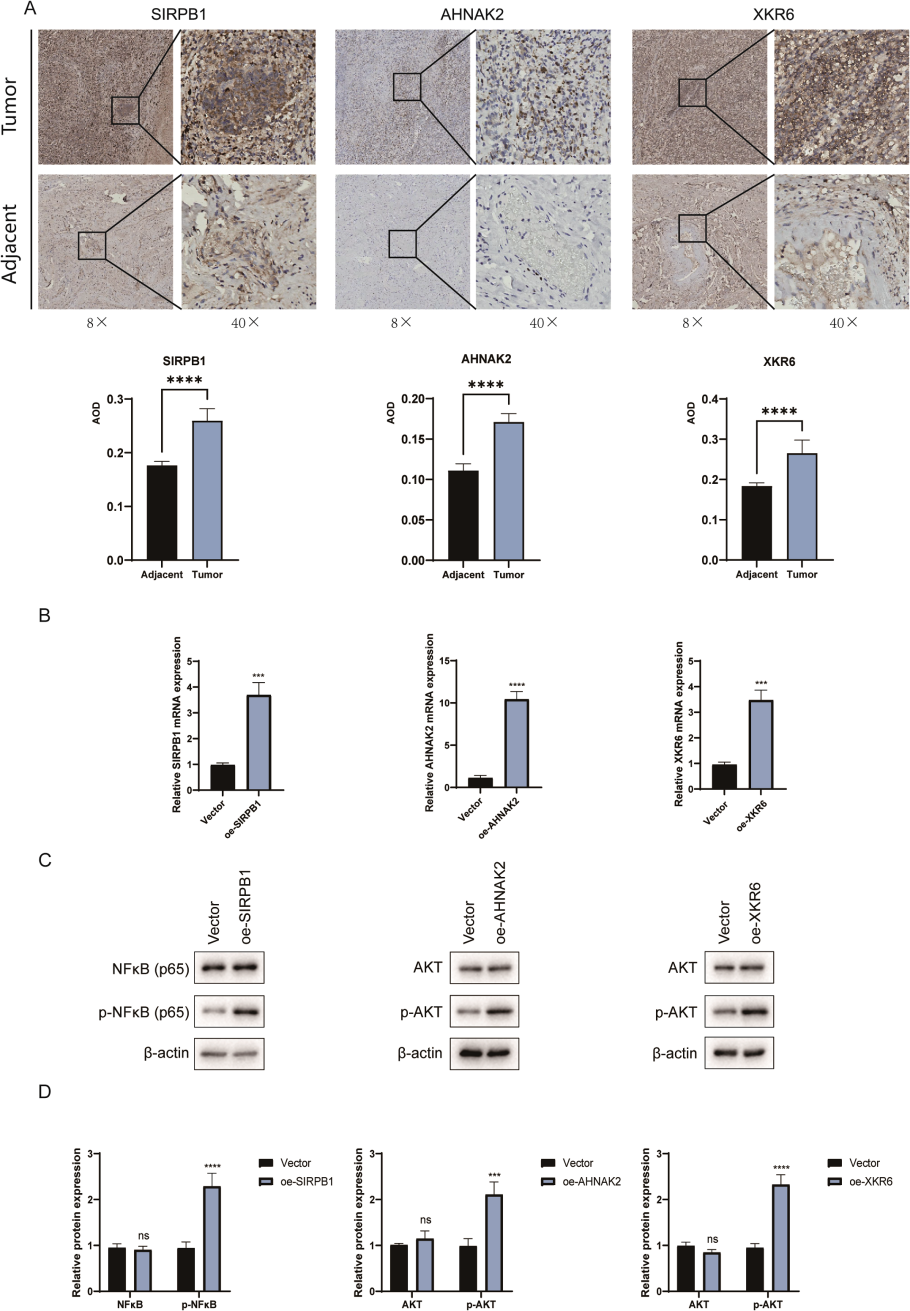
Effects of copy number variation (CNV) on *SIRPB1*, *AHNAK2*, and *XKR6* gene expression and cellular pathway functionality. **(A)** The expression of SIRPB1, AHNAK2, and XKR6 proteins in cancerous and adjacent tissues of patients with PMMC. **(B–D)** Effects of increased SIRPB1, AHNAK2, and XKR6 expression on the activation of NFκB and AKT signaling pathways in HMV-II cells. *, p < 0.05; **, p < 0.01; ***, p <0.001; ****, p < 0.0001.

We found increased CNVs in *SIRPB1*, *AHNAK2*, and *XKR6* in patients with PMMC and further evaluated their impact on gene expression and cellular pathways. Immunohistochemical staining of sections from patients with PMMC revealed statistically significant increases in expression of SIRPB1, AHNAK2, and XKR6 in tumor-associated regions compared with adjacent tissue (**Figure 7A**). Subsequently, we transfected HMV-II cells with *SIRPB1*, *AHNAK2*, and *XKR6* genes. qPCR results demonstrated that, compared with the vector group, the expression levels of oe-*SIRPB1* (P = 0.0007), oe-*AHNAK2* (P < 0.0001), and oe-*XKR6* (P = 0.0004) in HMV-II cells were significantly increased (**Figure 7B**). Finally, we found that when *SIRPB1*, *AHNAK2*, and *XKR6* were overexpressed, p-NFκB and p-AKT were activated in the SIRPB1 (P < 0.0001), AHNAK2 (P = 0.0001), and XKR6 groups (P < 0.0001), respectively, compared with their levels in the vector group (**Figures 7C, D**). However, there were no statistically significant differences in *NFκB* and *AKT* levels between the SIRPB1, AHNAK2, and XKR6 groups and the vector group, respectively (**Figures 7C, D**).

## 4 Discussion

In our research, somatic SNVs and somatic InDels were found mainly in CDS and intronic regions. The Somatic SNV CDS area is mostly synonymous with SNPs and missense SNPs, and the Somatic INDEL CDS area is primarily frameshift deletion and frameshift insertion. A study by Govindan et al. has shown that non-smokers and light-smokers with non-small cell lung tumors (NSCLC) C>T dominate (35); Alexandrov et al. found that the most mutational feature is the reaction of 5-methylcytosine deamination at CpG sites C> T, which is related to the patient’s age, most of which existed before the tumor occurred (36). At the same time, C>T/G>A, that is, cytosine deamination to uracil, is dominant in patients with PMMC.

Next, we used the NMF method to decompose point mutations into multiple different mutation features, clustered them with the mutation features in the COSMIC database, and used the annotation information of known features similar to the sample mutation features to explain the sample mutation process. Our study found that the PMMC mutation features SBS5 and SBS40. The research by Apostolos et al. on SKCM showed that SKCM has high cosine similarity with SBS7a/SBS7b, SBS5, and SBS1. The number of mutations in SBS5 and SBS1 is related to aging and shows similar clock characteristics, whereas SBS7a/b features are similar to SBS1 and SBS5. Previous studies (37) have reported that SBS40 mutation features were found in the open chromatin area of prostate cancer (36), and there are few reports related to melanoma (38). Because PMMC originates in the cervical mucosa, its microenvironment primarily comprises cervical epithelial cells, stromal cells, and immune cells (39). In contrast, SKCM originates in the epidermis, and its microenvironment includes keratinocytes, fibroblasts, immune cells, and skin appendage structures (40). This makes PMMC more susceptible to the local cervical mucosal immune system, while SKCM is primarily associated with the cutaneous immune system. Although the mutational profile of SBS5 in PMMC and SKCM are similar, SBS5/SBS40 is constrained by different tissue-specific environments, potentially leading to distinct mutational processes and pathogenic mechanisms.

Juliette et al. (41) reported 20 susceptibility genes for melanoma, *p16INK4A* and *p14ARF* (located at the same locus, *CDKN2A*), *CDK4*, *BAP1*, *RAD51B*, *POLE*, *TERT*, *POT1*, etc. Surprisingly, the CPGs identified in our PMMC study were *ATRX*, *BPTF*, *NTRK1*, *NCOR2*, etc., which are different from melanoma susceptibility genes. In mucosal melanoma, ATRX inactivation causes telomere prolongation, induces cell immortality, and facilitates disease onset (42–44). Bromodomain PHD finger transcription factor (BPTF) promotes melanoma development by regulating B-cell lymphoma-2 (BCL2) anti-apoptosis, influencing melanocyte stem cell differentiation, and affecting melanoma proliferation via extracellular signal-regulated kinases regulation (45–47). The fusion of neurotrophic tyrosine kinase type 1 is rare in melanoma but common in other tumors, possibly regulating MAPK/phosphoinositide 3-kinase (PI3K) pathways (48), and its mutation marks Spitz melanoma (49). Nuclear receptor corepressor 2 rs2342924 T>C predicts SKCM prognosis, likely through Notch pathway mutations, that increase melanoma cell proliferation (50–54).

To further understand the process of PMMC, this study identified the driving genes, *TP53*, *NRAS*, *EPHA3*, *SMYD4*, *ATRX*, *APC*, etc. Previous studies have confirmed that *TP53*, *NRAS*, *EPHA3*, *ATRX*, and *APC* are the driver genes of melanoma. In our study, considering the somatic SNV and InDel mutations, using MuSiC software, we found that the SMG in PMMC is *AHNAK2*, *SIRPB1*, *XKR6*, *ATAD3A*, *TP53*, *AKT3*, and *SMYD4*. AHNAK2 (55), XKR6 (56, 57), TP53 (58), and AKT3 (59) are associated with melanoma. Likewise, TCGA and COMIC databases showed that TP53 and AHNAK2 are associated with melanoma. However, no relationship between SIRPB1, ATAD3A, and SMYD4 and melanoma was observed. SMG-related protein-protein interaction network shows that WDR20, AR, AKT3, TP53, CCDC6, SMYD4, and ATAD3A interact, and the related signaling pathways are mainly concentrated in the MAPK signaling pathway, cGMP-PKG signaling pathway, Human T-cell leukemia virus 1 infection, cellular senescence, oxytocin signaling pathway, Wnt signaling pathway, and the B cell receptor signaling pathway. Building on this, we further verified at the cellular level that P53, AKT3, SMYD4, and ATAD3A promote melanoma cell proliferation and invasion, and found that P53 forms an interaction network with AKT3, SMYD4, and ATAD3A, suggesting a crucial role of AKT3, P53, SMYD4, and ATAD3A in PMMC. Furthermore, the tumor microenvironment (TME) plays a crucial role in cancer progression, whose importance is comparable to that of the tumor origin tissue. Comparing the mutation profiles of PMMC, the TCGA cohort (skin melanoma and cervical cancer), and the COSMIC cohort (skin melanoma), we found that *DSCAML1* is the commonly mutated gene in both CC and PMMC. As a member of the cell adhesion molecule family, *DSCAML1* is primarily involved in processes such as cell-cell adhesion, signal transduction, and neural development (60, 61). Its mutations may participate in TME remodeling by influencing cell-cell interactions (altering the adhesion ability of tumor cells to stromal cells and immune cells, thereby affecting tumor cell migration, invasion, and immune escape), regulating immune cell infiltration and function (affecting chemokine or cytokine expression, recruiting more immunosuppressive cells, or inhibiting antitumor immune responses), and participating in signaling pathway regulation (abnormally activating or inhibiting pathways related to tumor progression such as PI3K/AKT and MAPK, affecting tumor cell proliferation, apoptosis, metabolism, and immunogenicity).

Finally, to further elucidate chromosomal changes in PMMC, somatic CNV analysis was performed. Both PMMC and TCGA SKCM datasets revealed 33 overlapping CNV genes. Notably, PMMC exhibited CNV differences in 5,712 genes compared to TCGA, with three specific CNV genes and SMGs, namely *SIRPB1*, *AHNAK2*, and *XKR6*. *AHNAK2* is expressed in all muscle cells, and its encoded protein interacts with S100B and other proteins to participate in cell structure and calcium signal transduction, and is related to melanoma (55). In generalized epilepsy syndrome and absence seizures, *XKR6* is deleted and may be affected by CNV (62). Zhang et al. confirmed XKR6 mutations across melanoma-related transport proteins and signaling pathways, linking these to biological processes (56). We further used cell models and clinical pathology to verify that as CNV increased in patients with PMMC, the expression levels of SIRPB1, AHNAK2, and XKR6 also increased; furthermore, SIRPB1, AHNAK2, and XKR6 may exert their effects by affecting the NFκB and AKT signaling pathways.

Previous studies have shown that the most frequently mutated genes in MM (>10%) include neurofibromin 1 (*NF1*), which negatively regulates the RAS/MAPK signaling pathway; the KIT proto-oncogene receptor tyrosine kinase (*KIT*), which is involved in promoting cell proliferation; and splicing factor 3b subunit 1 (*SF3B1*), which drives aberrant RNA splicing (3, 63, 64). Genes with lower mutation frequencies (5–10%) include neuroblastoma RAS viral oncogene homolog (*NRAS*), which maintains pro-survival signaling; sprouting-related EVH1 domain protein 1 (*SPRED1*), an inhibitor of RAS pathway activation; ATP-dependent helicase (*ATRX*), which is essential for maintaining genomic stability; and the B-Raf proto-oncogene (*BRAF*), which promotes cell growth (65). Notably, the *MITF* gene, a regulator of melanocyte development and differentiation, exhibits a dual mutational pattern: in vulvar melanomas, it rarely harbors the germline variant E318K, a well-documented risk factor for cutaneous melanoma, but is frequently amplified in several MM subtypes, suggesting a context-dependent role in tumorigenesis (64, 66). Our study also found *TP53* and *AKT3* mutations in patients with PMMC. *TP53* and *AKT3* are directly involved in the regulation of the cell cycle and are associated with abnormalities in signal transduction. Furthermore, CNV mutations increase the expression of *SIRPB1*, *AHNAK2*, and *XKR6*. These genes can also activate the NFκB and AKT signaling pathways, further affecting tumor cell proliferation and metastasis. However, our study also revealed some mutational profiles that differ from those in MM. *SMYD4* (67) and *ATAD3A* (68) mutations have been reported in patients with PMMC. Mutations in *SMYD4* may affect gene expression by altering chromatin states, while mutations in *ATAD3A* may disrupt mitochondrial homeostasis and affect energy metabolism. This suggests that PMMC patients may not only have abnormalities in signal transduction and tumor suppression but also imbalances in multi-level regulatory networks influenced by epigenetic modifications and mitochondrial dysfunction.

In recent years, immunotherapy has revolutionized the treatment of advanced melanoma (69). However, the response rate to anti-PD-1/PD-L1 therapy in MM is lower than in patients with cutaneous melanoma (70). These findings have prompted further research into the mechanisms of immunotherapy resistance. Studies have shown that *TP53* mutations can enhance sensitivity to PD-1 inhibitors in two ways: first, mutations lead to DNA repair defects, significantly increasing tumor mutational burden (TMB), increasing neoantigen presentation, and activating T cell-mediated immune responses. Second, some *TP53* mutations can activate programmed death-ligand 1 (PD-L1) expression through the NF-κB pathway, resulting in an immune escape phenotype (71). However, truncating *TP53* mutations (such as nonsense mutations) may completely abolish transcriptional regulation, leading to decreased tumor mutation burden (TMB) or loss of PD-L1 expression, which in turn predicts immunotherapy resistance (72). Buchbinder et al. found that despite the high prevalence of *TP53* mutations in patients with MM, mutations in genes such as *SF3B1*/*KIT*/*NF1* were not significantly associated with ICB response, suggesting the need for a comprehensive assessment combining TMB, PD-L1 expression, and lactate dehydrogenase (LDH) levels (73). *AKT3*, a core member of the PI3K-AKT-mTOR pathway, can inhibit immune responses through multiple pathways: promoting the infiltration of regulatory T cells (Tregs), inhibiting the maturation of dendritic cells (DCs), and reducing the cytotoxicity of tumor-infiltrating lymphocytes. In clinical trials for MM, the response rate (RR) of monotherapy with PD-1 inhibitors is only approximately 23.5%, partly attributed to the immunosuppressive state caused by persistent activation of the PI3K-AKT pathway (74). Combining AKT inhibitors with PD-1 inhibitors may improve the efficacy of PD-1 inhibitors by reversing Treg infiltration and enhancing CD8+ T cell function (75). In addition, mutations in *SIRPB1*, a signaling regulator, may affect macrophage phagocytosis. *AHNAK2* and *XKR6* are involved in cell adhesion and signal transduction, potentially regulating interactions between tumor cells and immune cells. In multiple myeloma, CTLA-4 inhibitors enhance effector T cell function by blocking early T cell activation signals, but this is associated with severe immune-related toxicities (such as colitis and hepatitis). *SIRPB1* mutations may regulate the balance between efficacy and toxicity of cytotoxic T-lymphocyte-associated protein 4-4 inhibitors by affecting macrophage polarization (76), but this mechanism requires further analysis through single-cell sequencing and spatial transcriptomics.

However, our study has limitations. First, the sample size in this study was small, including only three patients with PMMC, which may indeed affect the robustness and generalizability of the study results. We will actively collaborate with other research institutions in future studies to characterize the molecular pathological features of PMMC patients more comprehensively. In addition, we performed WES on PMMC patients and subsequently combined mRNA transcriptomics, proteomics, metabolomics, and other methods to elucidate the pathogenesis of PMMC and possible therapeutic targets from multiple perspectives. Finally, our study is based on WES, cell experiments, and clinical validation of small samples, which may limit direct clinical inference. These findings need to be verified based on larger clinical samples.

## 5 Conclusion

In this study, by analyzing the mutation spectrum and mutation characteristics, we found that C>T/G>A is dominant in PMMC, and the mutation characteristics are closer to SBS5 and SBS40 than those in SKCM. Further analysis of frequently mutated genes in PMMC, combined with *in vitro* assays and co-IP validation, revealed that P53 interacts with AKT3, SMYD4, and ATAD3A, potentially implicated in PMMC pathogenesis. Finally, SMG and CNV analyses, combined with integrated analysis with the TCGA and COMIC databases, revealed that copy number gains may regulate the expression of *SIRPB1*, *AHNAK2*, and *XKR6*, thereby activating the NFκB and AKT signaling pathways, further impacting the development and progression of PMMC. In summary, we describe the commonality of PMMC with melanoma reported previously in public databases and the literature, as well as unique mutant genes. We hope that through this research, PMMC-related molecular markers can be explored, personalized diagnosis and treatment plans can be provided to patients with PMMC, and the prognosis of patients can be improved.

## Data Availability

The original contributions presented in the study are included in the article/supplementary material. Further inquiries can be directed to the corresponding author.

## Supplementary material

Supplementary Figure 1 Cosine similarity heat map of the mutation feature. The cosine similarity between the sample mutation feature and 76 known mutation features is presented here. The darker the color, the closer the cosine value to 1, and the higher the feature similarity, the more likely it is that the same feature. Above the picture is the use of unsupervised hierarchical clustering, cluster analysis of the sample mutation characteristics, and 67 known mutation characteristics.

Supplementary Figure 2 The Cancer Genome Atlas (TCGA) database cervical cancer (CC) SNP waterfall chart.

Supplementary Figure 3 The COMIC, TCGA database (melanoma and cervical cancer), and SMG Venn diagram of patients with PMMC. Cervical cancer, CC.

Supplementary Figure 46 CNV analysis of PMMC patients. Circos display of CNV of patients with PMMC. Red means an increase in copy number, blue means a missing copy number, and green means a normal copy number.

Supplementary Table 1 Clinical characteristics of the three patients.

Supplementary Table 2 The coding sequences of *SIRPB1*, *AHNAK2*, and *XKR6*.

Supplementary Table 3 qPCR primer sequences of *GAPDH*, *P53*, *AKT3*, *SMYD4*, *ATAD3A*, *SIRPB1*, *AHNAK2*, and *XKR6*.

Supplementary Table 4 Alignment rate and coverage statistics of the whole exome sequencing of PMMC.

Supplementary Table 5 Results of screening of susceptibility genes in tissues adjacent to PMMC tissues.

Supplementary Table 6 Mutation feature screening annotation table.

Supplementary Table 7 Known driver gene analysis results.

Supplementary Table 8 Results of MuSic high-frequency mutation analysis in patients with PMMC.

Supplementary Table 9 Enrichment analysis results of metabolic pathways of high-frequency mutation genes.

Supplementary Table 10 Somatic copy number variation (CNV) statistics.
