# Supplementary figures and images for: The genetic landscape of primary malignant melanoma of the cervix using integrated bioinformatics analysis and whole-exome sequencing
Source: Front Oncol. 2025 Dec 17;15:1597153. doi: 10.3389/fonc.2025.1597153 (PMC12753427; doi:10.3389/fonc.2025.1597153)

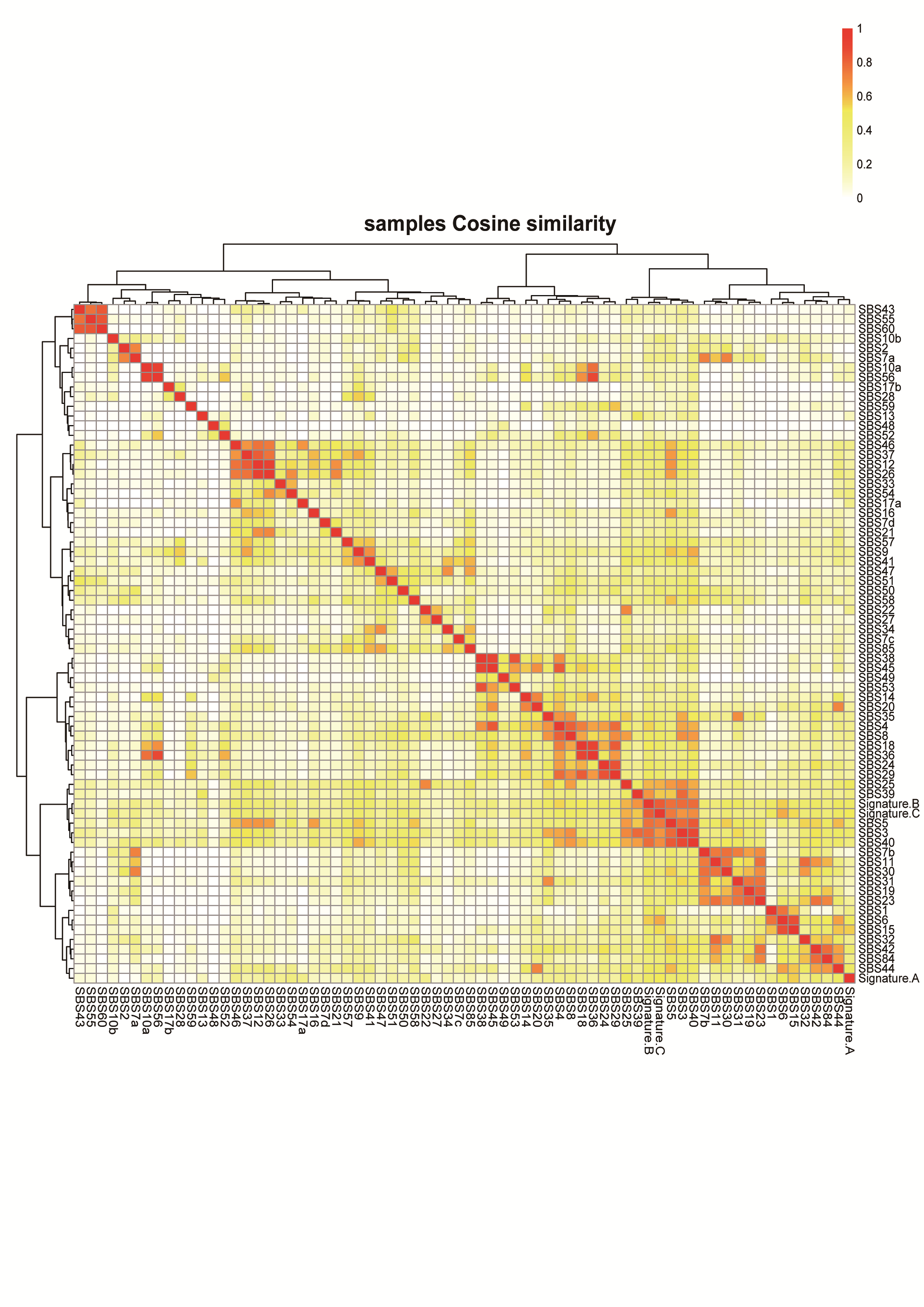

Supplement: Supplementary Figure 1 — Cosine similarity heat map of the mutation feature. The cosine similarity between the sample mutation feature and 76 known mutation features is presented here. The darker the color, the closer the cosine value to 1, and the higher the feature similarity, the more likely it is that the same feature. Above the picture is the use of unsupervised hierarchical clustering, cluster analysis of the sample mutation characteristics, and 67 known mutation characteristics. [file Image1.tif]

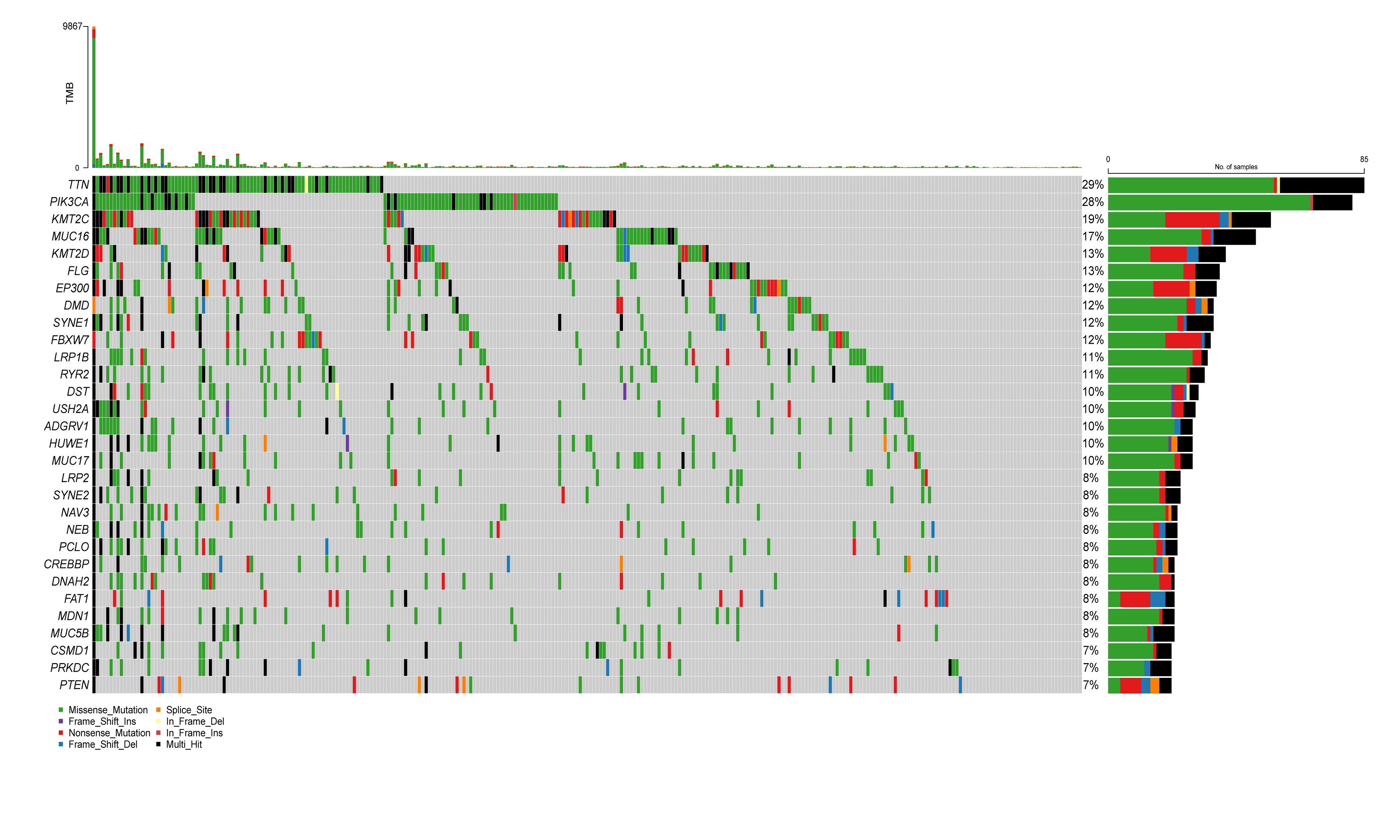

Supplement: Supplementary Figure 2 — The Cancer Genome Atlas (TCGA) database cervical cancer (CC) SNP waterfall chart. [file Image2.tif]

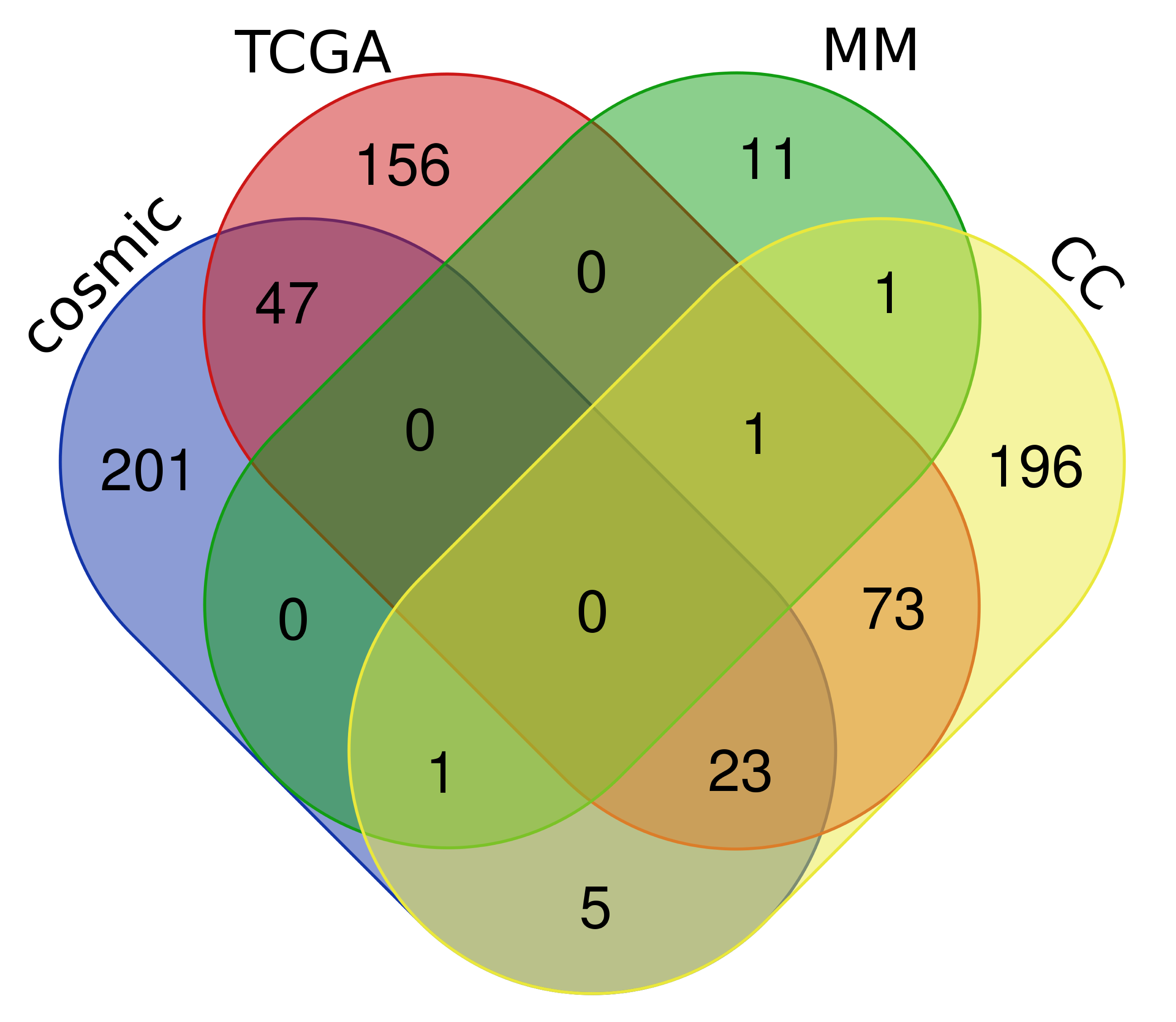

Supplement: Supplementary Figure 3 — The COMIC, TCGA database (melanoma and cervical cancer), and SMG Venn diagram of patients with PMMC. Cervical cancer, CC. [file Image3.tif]

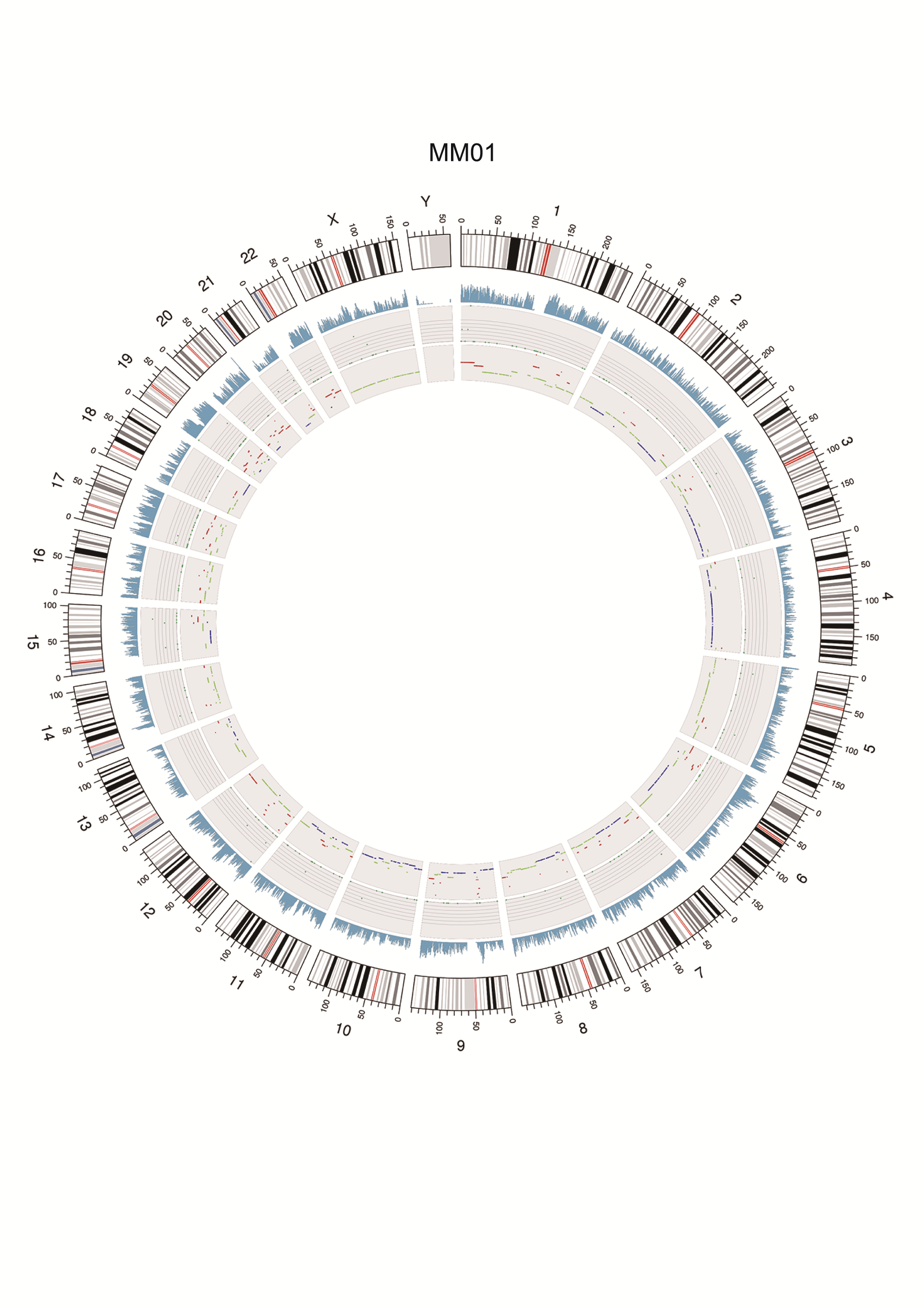

Supplement: Supplementary Figure 46 — CNV analysis of PMMC patients. Circos display of CNV of patients with PMMC. Red means an increase in copy number, blue means a missing copy number, and green means a normal copy number. [file Image4.tif]

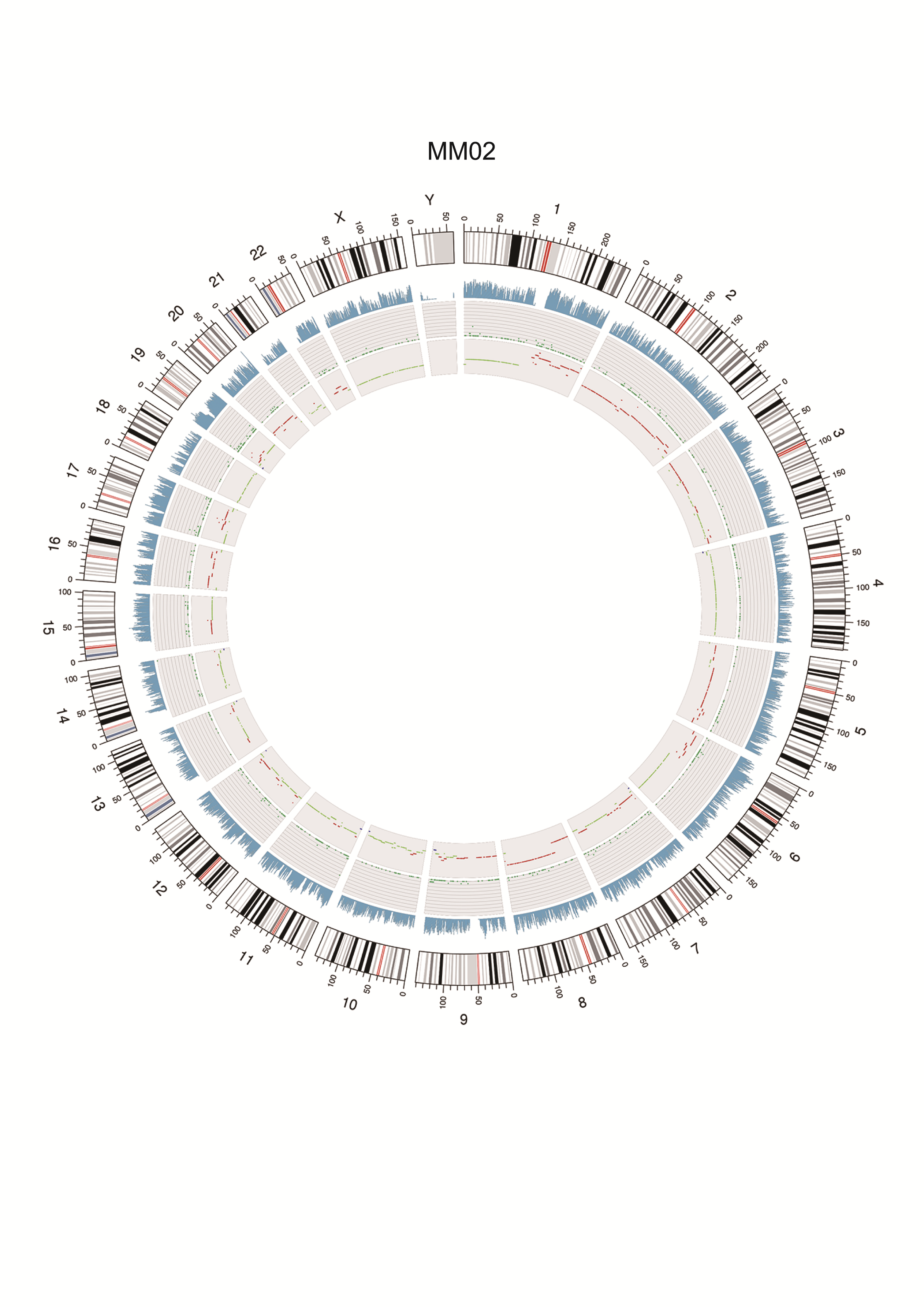

Supplement: Supplementary file 15 [file Image5.tif]

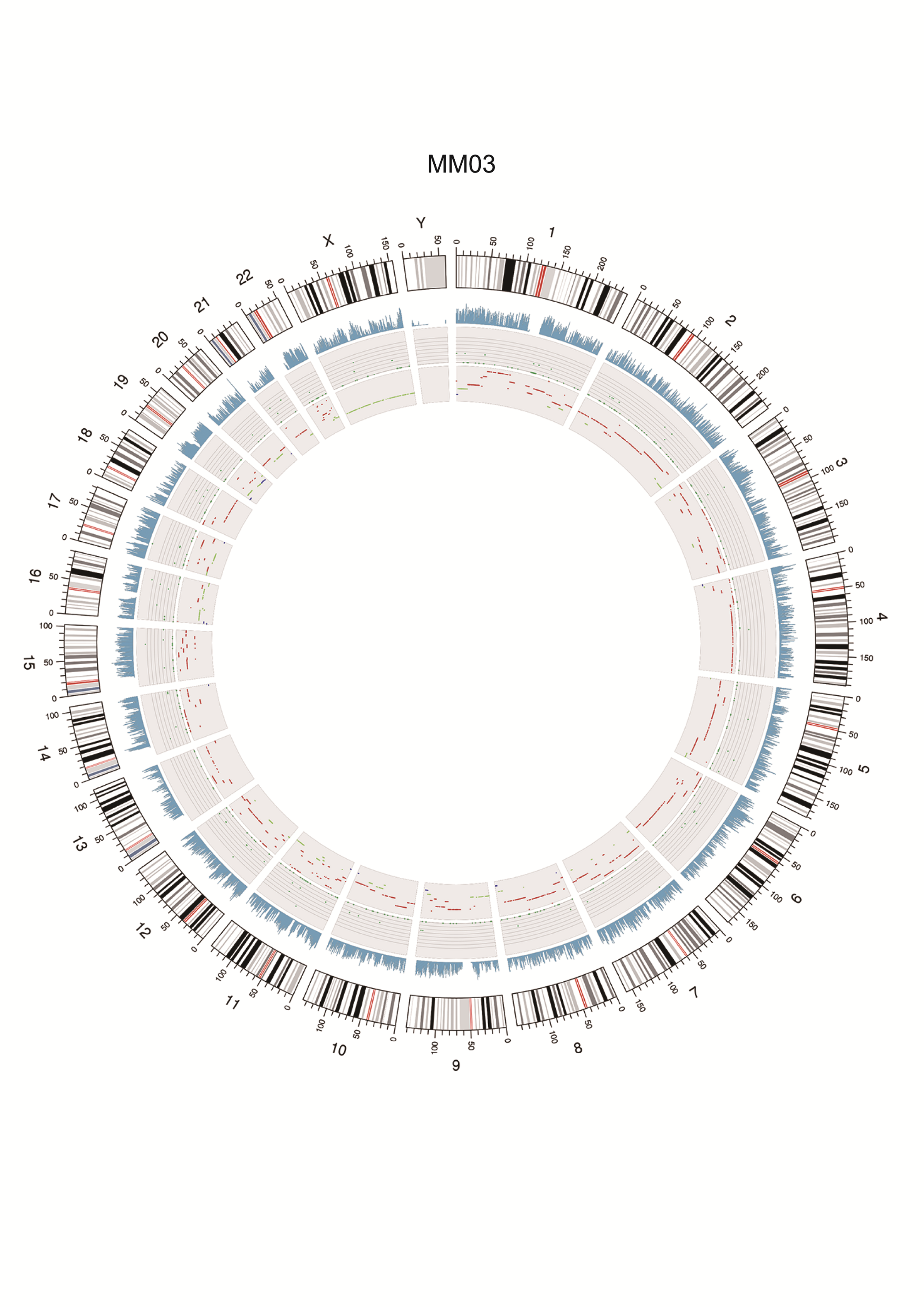

Supplement: Supplementary file 16 [file Image6.tif]
